# A *Vibrio vulnificus* VvpM Induces IL-1β Production Coupled with Necrotic Macrophage Death via Distinct Spatial Targeting by ANXA2

**DOI:** 10.3389/fcimb.2017.00352

**Published:** 2017-08-11

**Authors:** Sei-Jung Lee, Young Hyun Jung, Jun Sung Kim, Hyun Jik Lee, Sang Hun Lee, Kyu-Ho Lee, Kyung Ku Jang, Sang Ho Choi, Ho Jae Han

**Affiliations:** ^1^Department of Pharmaceutical Engineering, Daegu Haany University Gyeongsan, South Korea; ^2^Department of Veterinary Physiology, College of Veterinary Medicine, Research Institute for Veterinary Science and BK21 PLUS Program for Creative Veterinary Science Research Center, Seoul National University Seoul, South Korea; ^3^Medical Science Research Institute, Soonchunhyang University Seoul Hospital Seoul, South Korea; ^4^Departments of Biochemistry, Soonchunhyang University College of Medicine Cheonan, South Korea; ^5^Department of Life Science, Sogang University Seoul, South Korea; ^6^National Research Laboratory of Molecular Microbiology and Toxicology, Department of Agricultural Biotechnology, Center for Food Safety and Toxicology, Seoul National University Seoul, South Korea

**Keywords:** *V*. *vulnificus*, VvpM, macrophage, cell death, colonization, ANXA2, Atg5, NLRP3

## Abstract

An inflammatory form of phagocyte death evoked by the Gram-negative bacterium *Vibrio (V.) vulnificus* (WT) is one of hallmarks to promote their colonization, but the virulence factor and infectious mechanism involved in this process remain largely unknown. Here, we identified extracellular metalloprotease VvpM as a new virulence factor and investigated the molecular mechanism of VvpM which acts during the regulation of the inflammatory form of macrophage death and bacterial colonization. Mutation of the *vvpM* gene appeared to play major role in the prevention of IL-1β production due to *V. vulnificus* infection in macrophage. However, the recombinant protein (r) VvpM caused IL-1β production coupled with necrotic cell death, which is highly susceptible to the knockdown of annexin A2 (ANXA2) located in both membrane lipid and non-lipid rafts. In lipid rafts, rVvpM recruited NOX enzymes coupled with ANXA2 to facilitate the production of ROS responsible for the epigenetic and transcriptional regulation of NF-κB in the IL-1β promoter. rVvpM acting on non-lipid rafts increased LC3 puncta formation and autophagic flux, which are required for the mRNA expression of *Atg5* involved in the autophagosome formation process. The autophagy activation caused by rVvpM induced NLRP3 inflammasome-dependent caspase-1 activation in the promoting of IL-1β production. In mouse models of *V. vulnificus* infection, the *VvpM* mutant failed to elevate the level of pro-inflammatory responses closely related to IL-1β production and prevented bacterial colonization. These findings delineate *VvpM* efficiently regulates two pathogenic pathways that stimulate NF-κB-dependent IL-1β production and autophagy-mediated NLRP3 inflammasome via distinct spatial targeting by ANXA2.

## Introduction

One of the critical activities during bacterial colonization is killing phagocytes recruited by bacterial pathogens at the inflammation site (Fink and Cookson, 2007). *Vibrio vulnificus* is an extremely virulent anaerobic Gram-negative marine bacterium that often causes acute inflammatory responses and the killing of phagocytes in the gut (Toma et al., 2010; Lo et al., 2011). However, the underlying cellular mechanisms involved in this process remain largely undescribed. The majority of the virulence effects of *V. vulnificus* are reported to be derived from secreted toxins that are encoded by cytolytic pore-forming hemolysin (*VvhA*) (Jeong and Satchell, 2012; Song et al., 2016), multifunctional autoprocessing repeats in the toxin (*RTX*) (Lee et al., 2007; Jeong and Satchell, 2012), and elastase (*VvpE*) (Lee et al., 2015a,b, 2016). Several other secreted and cell-associated factors have also been proposed as potential virulence determinants that are also involved in the pathogenesis of *V. vulnificus*. A 55-kDa zinc-metalloprotease designated as *V. vulnificus* VvpM is considered to be another major exoprotease that causes cytotoxic effects and an autophagic process affecting intestinal epithelial cells (Lee M. A. et al., 2014, 2015). However, it remains unclear whether VvpM is a functional virulence factor of *V. vulnificus* specific to the inflammatory form of phagocyte death with the ability to promote bacterial colonization.

Interaction with a distinct host plasma membrane organized into lipid rafts and non–lipid rafts has been proposed as a highly evolved bacterial infectious stratagem to manipulate a wide range of host signaling events, including the inflammatory form of phagocyte death (Manes et al., 2003; Schroeder and Hilbi, 2007). This bacterial infection-induced spatial segregation of proteins into distinct membrane phases has been shown to circumvent specific innate host defenses that are crucial for infection propagation (Manes et al., 2003; Riethmuller et al., 2006). For instance, *Helicobacter pylori* vacuolating toxins (Fassino et al., 2002) and the entero toxin *Clostridium perfringens* (van der Goot and Harder, 2001) interact with a detergent-resistant cellular membrane composed of relatively abundant cholesterol as an initial attachment platform, thereby having inflammatory and cytotoxic effects on intestinal physiological functions. Hence, identifying the functional mechanism by which pathogens can evade the immune system by exploiting host-signaling cascades via distinct membrane domains may serve as a novel target for the treatment and/or prevention of infectious diseases.

The stimulation and release of pro-inflammatory cytokines from macrophages are critical steps in the activation of an effective innate host defense and subsequently for the modulation of adaptive immune responses (Netea et al., 2010). Interleukin (IL)-1β, a pro-inflammatory cytokine, is intensely produced by activated macrophages and has a central role in the regulation of many inflammatory forms of cell death typically triggered by inflammasome-mediated caspase-1 activation during bacterial infection (Schroeder and Hilbi, 2007; Lamkanfi et al., 2011; Guo et al., 2015). Aberrant production of active IL-1β from phagocytes is functionally associated with tissue damage and chronic inflammation and therefore should be tightly controlled by the innate host defense system (Lamkanfi et al., 2011). Autophagy is an essential innate host defense mechanism against pathogens that also promotes IL-1β production (Yuan et al., 2012; Kirienko et al., 2015). Although autophagy maintains cellular homeostasis and protects the host cell from harmful stimuli, autophagic cell death occurs when the cell is overwhelmed by infection or when apoptosis is inhibited (Labbe and Saleh, 2008). Notably, it has been shown that autophagy contributes to caspase-independent macrophage death (Xu et al., 2006). This suggests that autophagic cell death of macrophages is a predominant mechanism for controlling cell viability in the absence of apoptosis. On the other hand, several studies have been conducted to determine the factors that regulate IL-1β production, including lipid rafts (Oakley et al., 2009), transcription factors (Lee et al., 2015a), and methylation status (Hashimoto et al., 2009). Specifically, a recent report showed that many enteric bacterial pathogens, such as *Salmonella typhimurium* (Paesold et al., 2002; Jones et al., 2008), *H. pylori* (Ki et al., 2008), and enteropathogenic *Escherichia coli* (EPEC) (Nougayrede and Donnenberg, 2004) can affect a diverse set of epigenetic factors such as DNA methylation and histone modification to regulate the selective activation or silencing of specific host genes (Takahashi, 2014). Thus, studies regarding the process leading to IL-1β production during pathogen-induced macrophage death are likely to be critical to uncovering the bacterial infection mechanisms that promote colonization.

In the present study, therefore, we investigate the molecular mechanism of VvpM action, which drives the transcriptional and epigenetic regulation of the inflammatory process and controls autophagic cell death during the production of IL-1β in RAW 264.7 murine macrophage cells.

## Materials and methods

### Chemicals

Fetal bovine serum (FBS) was purchased from BioWhittaker Inc. (Walkersville, MO, USA). The following antibodies were purchased: NOX2, Rac1, and ANXA2 antibodies (BD Biosciences, Franklin Lakes, NJ, USA); NCF1 antibody (LifeSpan Biosciences, Seattle, WA, USA); p-JNK, JNK, PKCα^Ser 657^, p-NF-κBp65, NF-κBp65, IL-1β p17, caspase-1 p10, and β-actin antibodies (Santa Cruz Biotechnology, Paso Robles, CA, USA); Horseradish peroxidase (HRP)-conjugated goat anti-rabbit and goat anti-mouse IgG antibodies (Jackson Immunoresearch, West Grove, PA, USA). 2′, 7′-dichlorofluorescein diacetate (CM-H_2_DCFDA) was obtained from Invitrogen (Carlsbad, CA, USA). Methyl-β-cyclodextrin (MβCD), lipopolysaccharide (LPS), adenosine 5′-triphosphate disodium salt hydrate (ATP), 3-methyladenine, bafilomycin A1, *N*-acetyl-l-cysteine (NAC), rotenone, and 5-azacytidine were purchased from Sigma Chemical Company (St. Louis, MO, USA). The concentrations of all of pharmacological inhibitors listed did not show any significant cytotoxic effects by themselves as confirmed by FACS analysis in each experiment. All other reagents were of the highest purity commercially available and were used as received.

### Cells

RAW 264.7 murine macrophage and Caco-2 human intestinal epithelial cells were purchased from American Type Culture Collection (ATCC, Manassas, VA, USA) and cultured at 37°C in 5% CO_2_ in RPMI-1640 and DMEM containing 10% FBS and antibiotics. These cell lines have previously been used to evaluate the function of virulence factors of *V. vulnificus* in regulation of pro-inflammatory process and cytotoxic effects (Lee et al., 2015a; Song et al., 2016).

### Host cells infection protocol and IL-1β ELISA

Cells (1 × 10^6^ cells/mL) grown to confluence in 6-well plates were incubated in serum/antibiotic-free media for 24 h. The bacterial suspensions in serum/antibiotic-free media were added to the cells at a multiplicity of infection (MOI) of 1, 5, and 10, after which the infected cells were incubated for 1 h. For IL-1β ELISA, co-culture supernatants were obtained and harvested by centrifugation at 4,000 × g for 10 min at 4°C. This was repeated once more with the resulting supernatant. The clarified supernatants were filtered through a 0.22 μM pore membrane to ensure the removal of any remaining bacterial cells and debris. The IL-1β concentration in the culture medium was quantified by an enzyme-linked immunosorbent assay (ELISA) (R&D systems, Minneapolis, MN, USA) according to the manufacturer's instructions.

### Bacterial strains, plasmids, and culture media

All *V. vulnificus* strains (MO6-24/O; WT, MO6-24/O Δ*rtxA*; RTX mut, MO6-24/O *vvpE*; VvpE mut, MO6-24/O Δ*vvhBA*; VvhA mut, and MO6-24/O Δ*vvpM*; VvpM mut) are isogenic and naturally resistant to polymyxin B (Table 1). Unless otherwise noted, *V. vulnificus* strains were grown in Luria Bertani (LB) medium supplemented with 2.0% (wt/vol) NaCl (LBS) at 30°C. All media components were purchased from Difco (Difco Laboratories Inc., Detroit, MI). *V. vulnificus* were grown to mid-log phase (A_600_ = 0.500) corresponding to 2 × 10^8^ CFU/mL and centrifuged at 6,000 × g for 5 min. The pellet was washed with phosphate buffered saline (PBS) and adjusted to desired colony-forming unit (CFU)/mL based on the A_600_ determined using a UV–VIS spectrophotometer (UV-1800, Shimadzu, Japan) to estimate culture density.

**Table 1:** Plasmids and bacterial strains used in this study.

**Strain or plasmid**	**Relevant characteristics^a^**	**Reference or source**
**BACTERIAL STRAINS**		
***V. vulnificus***		
MO6-24/O	Clinical isolate; virulent; WT	Laboratory collection
CMM111	MO6-24/O *vvpE*::pKC9844; elastase deficient; VvpE mut	Jeong et al., 2000
KK1408	MO6-24/O Δ*vvhBA*; VvhA mut	This study
ML05	MO6-24/O Δ*vvpM*; Km^r^ VvpM mut	Lee et al., 2013
MW064	MO6-24/O Δ*rtxA*::*nptI*; Km^r^; RTX mut	Lee et al., 2007
***E. coli***		
DH5α	λ^−^Φ80d*lacZ*ΔM15 Δ(*lacZYA-argF*)*U169 recA1 endA1 hsdR17* (${\text{r}}_{\text{K}}^{-}$ ${\text{m}}_{\text{K}}^{-}$) *supE44 thi-1 gyrA relA1*; plasmid replication	Laboratory collection
S17-1λ*pir*	λ-*pir* lysogen; *thi pro hsdR hsdM^+^ recA* RP4-2 Tc::Mu-Km::Tn7; Tp^r^ Sm^r^; host for π-requiring plasmids; conjugal donor	Simon et al., 1983
**PLASMIDS**		
pDM4	R6K γ *ori sacB*; suicide vector; *oriT* of RP4; Cm^r^	Milton et al., 1996
pKK1407	pDM4 with Δ*vvhBA*; Cm^r^	This study
pRK415	IncP *ori*, broad-host-range vector; *oriT* of RP4; Tc^r^	Keen et al., 1988
pKK1535	pRK415 with *vvpM*; Tc^r^	This study

a *Km^r^, kanamycin resistant; Tp^r^, trimethoprim resistant; Sm^r^, streptomycin resistant; Cm^r^, chloramphenicol resistant; Tc^r^, tetracycline resistant*.

### Generation of the exotoxin mutants

Various deficient mutants in *VvpM, RTX*, and *VvpE* genes were generated by the methods as described previously (Jeong et al., 2000; Lee et al., 2007). For generation of *VvhA* mutant (Milton et al., 1996), the *vvhBA* operon was inactivated *in vitro* by deletion of the open reading frame (ORF) of *vvhBA* (998-bp of 1,935-bp) using the PCR-mediated linker-scanning mutation method as described previously (Jang et al., 2016). Briefly, pairs of primers VVHBA01-F and -R (for amplification of the 5′ amplicon) or VVHBA02-F and -R (for amplification of the 3′ amplicon) were designed and used (Table S1). The *vvhBA* operon with 998-bp deletion was amplified by PCR using the mixture of both amplicons as the template and VVHBA01-F and VVHBA02-R as primers. The resulting Δ*vvhBA* was ligated into SpeI-SphI-digested pDM4 (Milton et al., 1996) to generate pKK1407. *Escherichia coli* S17-1 λ*pir, tra* strain (Simon et al., 1983) containing pKK1407 was used as a conjugal donor to either *V. vulnificus* MO6-24/O to generate the *VvhA* mutant KK1408 (Table 1).

### Complementation of the *VvpM* mutant and purification of the recombinant VvpM

To complement the *vvpM* mutation, an ORF of *vvpM* was amplified from the genomic DNA of *V. vulnificus* MO6-24/O by PCR with the primer pair VVPM001F and VVPM001R (Table S1) and then digested with BamHI and SacI. The amplified *vvpM* ORF was subcloned into the broad-host-range vector pRK415 (Keen et al., 1988) linearized with the same enzymes (Table 1) to result in pKK1535. *E. coli* S17-1 λ *pir, tra* strain (Simon et al., 1983) containing pKK1535 was used as a conjugal donor to *VvpM* mutant. The plasmid pKK1535 was delivered into the *VvpM* mutant by conjugation as described previously (Jang et al., 2016). Recombinant (r) VvpM protein (Lee M. A. et al., 2014; Lee et al., 2015a) was kindly provided by Prof. Kyu-Ho Lee (Sogang University, Korea). We have further checked the LPS contamination in rVvpM protein by using endotoxin quantitation kit (Pierce® LAL Chromogenic Endotoxin Quantitation Kit, Thermo Fisher Scientific Inc. Waltham, MA, USA). The level of endotoxin in 100 pg/mL of rVvpM was less than 0.003 EU. Thus, we suggest that rVvpM is a purified recombinant protein containing very low level of endotoxin and suitable for our experiments in this study.

### RNA isolation and reverse transcription-polymerase chain reaction (RT-PCR)

Total RNA was extracted using the RNeasy Plus Mini Kit (Quiagen, Valencia, CA, USA). Reverse transcription (RT) was carried out with 3 μg of RNA using a Maxime RT premix kit (iNtRON Biotechnology, Sungnam, Korea). β-Actin was used as an endogenous control. The cDNA (5 μL) for *Annexins* family were amplified.

### Quantitative real-time polymerase chain reaction (qRT-PCR)

The real-time quantifications of pro-inflammatory cytokines and autophagy related genes were performed using a Rotor-Gene 6000 real-time thermal cycling system (Corbett Research, New South Wales, Australia) with a QuantiMix SYBR Kit (PhileKorea Technology, Daejeon, Korea) according to the manufacturer's instructions. β-Actin was used as an endogenous control.

### Flow cytometry

Cells were synchronized in the G_0_/G_1_ phase by culture in serum-free media for 24 h before incubation of rVvpM The necrotic cell death was detected with an Annexin V and PI staining kit (BD Biosciences, Franklin Lakes, NJ) according to the manufacturer's instructions. Briefly, the cells were detached with 0.05% trypsin/EDTA and 1 × 10^5^ cells were resuspended with Annexin V binding buffer. And then the cells were stained with Annexin V (25 μg/ml) and PI (125 ng/mL), and incubated for 15 min at room temperature in the dark. The sample was read by flow cytometry and analyzed using CXP software (Beckman Coulter, Brea, CA). Samples were gated to exclude debris (forward light scatter [FSC] area vs. side scatter-area), and then any cell doublets were excluded using FSC-area vs. FSC-width analysis.

### Apoptosis/necrosis detection

Cell death was also detected with an Apoptosis/Necrosis Detection kit (Abcame Cambridge, MA, USA) according to the manufacturer's instructions. Briefly, the cells were treated with Apopxin Green indicator as a phosphatidylserine marker and 7-aminoactinomycin D (AAD), and were then incubated in the dark for 60 min at room temperature. After the cells were rinsed with ice-cold PBS, the level of cell death was examined using a luminometer (Victor3; Perkin-Elmer, Waltham, MA) and quantified by measuring absorbance at excitation and emission wavelengths of 490 and 525 nm for detection of Apopxin Green Indicator or at excitation and emission wavelengths of 490 and 650 nm for detection of 7-AAD.

### Small interfering (si)RNA transfection

Cells were grown until 75% of the surface of the plate and transfected for 36 h with ON-TARGETplus siRNAs mixed by 4 different siRNAs specific for *ANXA2, cav-1, PKC*α, *JNK, NF*-κ*Bp65, Atg5*, and *NLRP3* (GE Dharmacon, Lafayette, CO, USA) or non-targeting (nt) siRNA as a negative control (GE Dharmacon, Lafayette, CO, USA) with HiPerFect Transfection Reagent (QIAGEN, Valencia, CA, USA) according to the manufacturer's instructions. The siRNA efficacy for *ANXA2, cav-1, PKC*α, *JNK, NF*-κ*Bp65, Atg5*, and *NLRP3* determined by Western blot was 71, 69, 76, 75, 69, 71, and 61%, respectively (Figure S1).

### Detergent-free purification of caveolin-rich membrane fraction

Cells were washed twice with ice-cold PBS, scraped into 2 mL of 500 mM sodium carbonate (pH 11.0), transferred to a plastic tube, and homogenized with a Sonicator 250 apparatus (Branson Ultrasonic, Danbury, CT) using three 20-sec bursts. The homogenate was adjusted to 45% sucrose by the addition of 2 ml 90% sucrose prepared in 2-(N-morpholino) ethanesulfonic acid (MES)-buffered solution consisting of 25 mM MES-buffer solution (pH 6.5) and 0.15 M NaCl and placed at the bottom of an ultracentrifuge tube. A 5–35% discontinuous sucrose gradient was formed (4 ml each of 5 and 35% sucrose, both in MES-buffer solution containing 250 mM sodium carbonate) and centrifuged at 40,000 × g for 20 h in a Beckman SW41 Rotor (Beckman Coulter, Fullerton, CA). Eight fractions were collected and analyzed by 12% SDS-PAGE.

### Immunofluorescence analysis

Cells were fixed in 4% paraformaldehyde in PBS for 10 min at room temperature, permeabilized in 0.1% Triton X-100 in PBS for 5 min, and blocked in PBS containing 5% (v/v) normal goat serum (NGS) for 30 min at room temperature. Samples were then stained with primary antibody for overnight at 4°C. Following three washes with PBS, the samples were incubated with Alexa 488-conjugated goat anti-rabbit/mouse IgM (Invitrogen Co., Carlsbad, CA, USA), and counterstained with PI in PBS containing 5% (v/v) NGS for 2 h. After washing with PBS, samples were mounted on slides and visualized with an Olympus FluoView™ 300 confocal microscope with 400x objective. The expressions of LC3 in immunofluorescence image was quantified by using Image J software (NIH, Bethesda, MD), which measures the stained area per microscopic filed with consistent threshold. On the other hand, the cell numbers showing the membrane translocation of PKCα and the nuclear translocation of p-NF-κB were directly counted per random microscopic filed and converted the numbers to a percentage by multiplying by 100. Ten random fields per coverslip were counted.

### Intracellular reactive oxygen species (ROS) detection

2′, 7′-dichlorofluorescein diacetate (CM-H_2_DCFDA) and MitoSOX™ Red Mitochondrial Superoxide Indicator (Thermo Fisher Scientific Inc.Waltham, MA, USA) were used to detect the intracellular and mitochondrial ROS production, respectively. To quantify the intracellular ROS levels, the cells treated with 10 mM CM-H_2_DCFDA were rinsed twice with ice-cold PBS and then scraped. A 100 μL cell suspension was loaded into a 96-well plate and examined using a luminometer (Victor3; Perkin-Elmer, MA, USA) and a fluorescent plate reader at excitation and emission wavelengths of 485 and 535 nm, respectively.

### Western blot analysis and subcellular fractionation

Western blotting was performed as previously described with minor modifications (Lee S. J. et al., 2014). Cells were harvested, washed twice with PBS, and lysed with buffer (20 mM Tris [pH 7.5], 1 mM EDTA, 1 mM EGTA, 1% Triton X-100, 1 mg/mL aprotinin, and 1 mM phenylmethylsulfonylfluoride [PMSF]) for 30 min on ice. The lysates were then cleared by centrifugation (22,250 × g at 4°C for 30 min). Equal amounts of protein (20 μg) were resolved by 10~15% sodium dodecyl sulfate polyacrylamide gel electrophoresis (SDS-PAGE) and transferred to a polyvinylidene fluoride (PVDF) membranes. The membranes were washed with TBST solution (10 mM Tris-HCl [pH 7.6], 150 mM NaCl, and 0.05% Tween-20), blocked with 5% skim milk for 1 h, and incubated with appropriate primary antibody at 4°C for overnight. The membrane was then washed and incubated with a horseradish peroxidase-conjugated secondary antibody for 2 h. The bands were visualized by enhanced chemiluminescence (Amersham Pharmacia Biotech Inc., Buckinghamshire, UK) and detected by using the Bio-rad ChemiDoc™ XRS+ System with images manipulated using the Image Lab™ software #170-8265 (Bio-Rad, Hercules, CA, USA). The subcellular fractionation method for the isolation of membrane and cytosolic proteins were previously reported (Cox and Emili, 2006).

### DNA binding activity of NF-κBp65

The DNA binding activity of NF-κBp65 was determined by a NF-κBP65 transcription assay kit (Cayman chemical, Ann Arbor, MI) according to the manufacturer's instructions. Briefly, double-stranded DNA (dsDNA) sequence containing NF-κB p65 response element was incubated with 2 mg/ml nuclear extracts. DNA-bound NF-κBp65 complex was captured by an immobilized antibody specific for NF-κBp65 and detected by addition of specific secondary antibody conjugated to HRP. The absorption was measured at 450 nm.

### Immunoprecipitation

Interaction of either Rac1 with ANXA2, NCF1, and caveolin-1 or ASC with NLRP3 was analyzed by immunoprecipitation and Western blotting. Cells were lysed with lysis buffer (1% Triton X-100 in 50 mM Tris–HCl pH 7.4 containing 150 mM NaCl, 5 mM EDTA, 2 mM Na_3_VO_4_, 2.5 mM Na_4_PO_7_, 100 mM NaF, 200 mM microcystin lysine–arginine, and protease inhibitors). Cell lysates (400 μg) were mixed with 10 μg of each antibodies. The samples were incubated for 4 h, mixed with Protein A/G PLUS-agarose immunoprecipitation reagent (Pierce, Rockford, IL, USA) and then incubated for an additional 12 h. The beads were washed four times, and the bound proteins were released from the beads by boiling in SDS-PAGE sample buffer for 5 min.

### Chromatin immunoprecipitation (ChIP)

ChIP was performed using the EZ-ChIP kit (EMD Millipore, Billerica, MA, USA) according to the manufacturer's protocol. Briefly, cells were treated with 1% formaldehyde for 15 min to cross-link proteins to DNA, lysed, and then sonicated. The lysate was incubated with primary antibodies overnight at 4°C. The immunocomplex was purified by incubation with 60 μL of protein G-agarose beads for 1 h and eluted for DNA purification. Quantitative real-time PCR was performed with primers for the *IL-1*β promoter flanking the putative NF-κBp65 binding sites. The following primer sequences of *IL-1*β promoter were used (sence and antisence respectively): −237 ~ −56 bp, 5′- TCCACCACGATGA CACACTT -3′ and 5′- GCTGTGAAATTTTCCCTTGG -3′. Anti-RNA polymerase II and normal mouse IgG were used as the positive and negative control for immunoprecipitation, respectively. The human *IL-1*β promoter sequence was found using the Eukaryotic Promoter Database. The putative binding sites were predicted using Alggen Promo software, version 3.0.2 (Messeguer et al., 2002; Farre et al., 2003).

### Methylation analysis

Genomic DNA from RAW264.7 cells was prepared with the QIAamp DNA Mini Kit (Qiagen, Valencia, CA, USA). The extracted DNA was treated with sodium bisulfite using EzWay™ DNA Methylation Detection Kit according to the manufacturer's instructions (KOMABIOTECH, Seoul, Korea). The methylation status of *IL-1*β gene was determined by methyl-specific PCR (MSP) analysis. We conducted MSP of *IL-1*β gene promoter containing the −328, −294, and −277 CpG sites due to their close distance to putative NF-κB binding sites. The following primer sequences were used (sense and antisense respectively): Methylated, 5′- TTTTAGTTTAAGTATAAGGAGGCGA-3′ and 5′-ACAC ATTCGCAAATATATCATCGTA-3′; Unmethylated, 5′-TTTTAGTTTAAGTATAAGGAGGTGA-3′ and 5′-ACACATTCACAAATATATCA TCATA -3′.

### Transmission electron microscopy (TEM)

Cells treated with rVvpM were fixed in Karnovsky's solution and 2% OsO_4_ in 0.1 M cacodylate at 4°C for 2 h, respectively. The cells were rinsed with distilled water briefly and stained with 0.5% uranyl acetate en bloc solution for overnight. After dehydration in graded ethanol series, samples were infiltrated with Spurr's resin. The samples were imaged by JEM1010 (JEOL, Tokyo, Japan) transmission electron microscope operating at 80 kV.

### Inflammasome RT^2^ profiler PCR array

The mouse Inflammasomes RT2 Profiler PCR Array (Qiagen, Valencia, CA, USA) was used to analyze gene expression of inflammasome components and signaling pathways in a cells treated with rVvpM for 24 h according to the manufacturer's instructions. In this array a set of optimized primer assays allows the detection of mRNA transcripts of 84 genes as well as five housekeeping genes in a 96-well plate by real-time PCR. Arrays were performed a minimum of three times for each treatment. Functional gene grouping profiles were provided as available online at http://www.sabiosciences.com/rt_pcr_product/HTML/PAMM-097Z.html#howitwork. PCR array data were analyzed using the GeneGlobe Data Analysis Center on QIAGEN's website at http://www.qiagen.com/kr/shop/genes-and-pathways/data-analysis-center-overview-page/.

### Mouse model and colonization assay

All animal procedures were performed following the National Institutes of Health Guidelines for the Humane Treatment of Animals, with approval from the Institutional Animal Care and Use Committee of Seoul National University (SNU-140108-4). Seven-week-old mice (*n* = 10) were received i.g. inoculation of boiled *V. vulnificus* (Cont.), *V. vulnificus* (WT), *VvpM* mutant, and VvpM complement at 1.3 × 10^9^ CFU/mL for 16 h and sacrificed. Half of ileum tissue was collected, washed, and homogenized. The homogenates of each organ were serially diluted and spread on LB agar containing polymyxin B (100 U/mL). CFUs were normalized to grams of intestinal tissues (CFU/g) to represent superficial bacterial counts. The other half were embedded in O.C.T. compound and stored at −70°C. Samples were then cut into 6-μm-thick frozen sections.

### Histologic damage score

Frozen sections of tissue samples were subjected to hematoxylin and eosin (H&E) staining for histological examinations. Histological parameters were determined in a blinded fashion by two experienced gastrointestinal pathologists as previously described (Clark et al., 2006). Briefly, scores were assigned as follows: 0 = no damage (normal); 1 = slight submucosal and/or lamina propria separation (mild); 2 = moderate separation of the submucosa and/or lamina propria and/or edema in the submucosa and muscular layers (moderate); 3 = severe separation of the submucosa and/or lamina propria and/or severe edema in the submucosa and muscular layers with regional villous sloughing (severe); or 4 = loss of villi and necrosis (necrosis). Intermediate scores of × 0.0 or × 0.5 were also used to more precisely assign the degree of intestinal damage.

### Ileal-ligated mouse model

To determine the functional role of VvpM we performed further experiment by using the ileal-ligated mouse model. Before surgery, mice were fasted for 24 h and anesthetized by intraperitoneal injection of a 2:1 mixture of Zoletil™ (20 mg/kg, Virbac Laboratories, Carros, France) and Xylazine HCl (10 mg/kg, Rompun®, Bayer, Germany). While maintaining the body temperature at 37°C using a heating pad, a small abdominal incision was made and a loop of middle ileum of intestine was isolated by silk suture (2–3 cm in length). The closed ileal loop was instilled with 100 μl of phosphate-buffered saline (PBS) containing WT, *vvpM* mutant, and *vvpM* complement at 1.3 × 10^9^ CFU/mL for 2 h. After putting the ileal loop back into the peritoneal cavity, the cavity was closed with suture. At 2 h after the inoculation of both strains, the mice were sacrificed and the intestinal loops were removed for real-time PCR and the hematoxylin and eosin (H&E) staining.

### Statistical analysis

Results are expressed as means ± standard errors (S.E.). All experiments were analyzed by ANOVA, followed in some cases by a comparison of treatment means with a control using the Bonferroni-Dunn test. Differences were considered statistically significant at *P* < 0.05.

## Results

### *V. vulnificus* metalloprotease VvpM induces IL-1β production coupled with necrotic cell death

**Figure 1:**
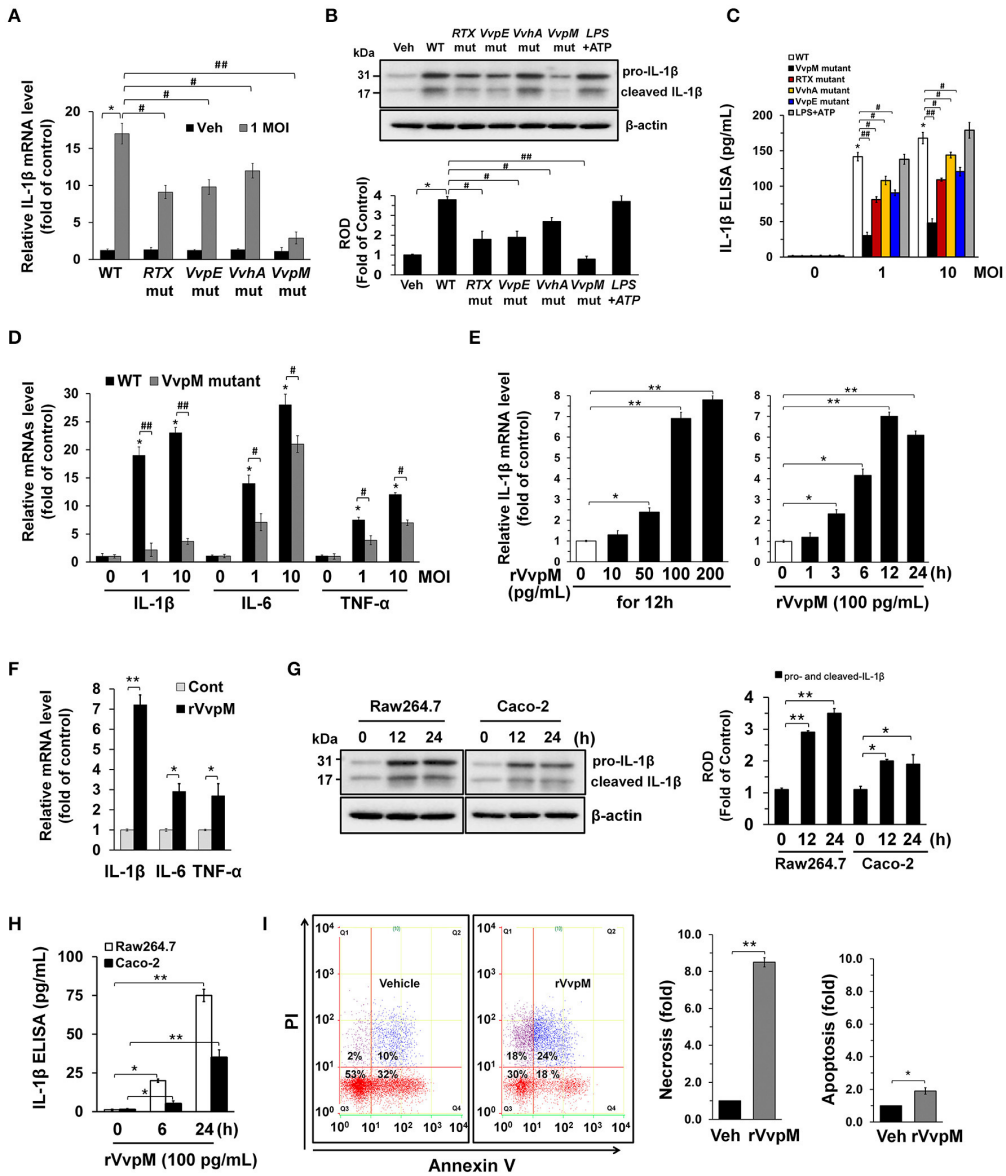
*V. vulnificus* metalloprotease VvpM induces IL-1β production coupled with necrotic cell death. The expression **(A)**, mature form **(B)**, and production **(C)** of IL-1β in RAW 264.7 murine macrophage cells infected with *V. vulnificus* (WT), various mutant deficients in *RTX, VvpE, VvhA*, and *VvpM* genes, and LPS (10 μg/mL) + ATP (1 mM) for 3 h are shown. Data represent the means ± S.E. (*n* = 5). **(D)** The expression of IL-1β, IL-6, and TNF-α in a cells infected with WT or *VvpM* mutant for 3 h is shown. *n* = 4. **(E)** Dose and time responses of rVvpM for 24 h in *IL*-*1*β mRNA expression are shown. *n* = 4. **(F)** The expression of IL-1β, IL-6, and TNF-α in a cells treated with rVvpM (100 pg/mL) for 12 h is shown. *n* = 3. **(G)** The levels of IL-1β mature form in RAW 264.7 and Caco-2 human intestinal epithelial cells treated with rVvpM (100 pg/mL) for 12 h was determined by Western blot. *n* = 3. **(H)** The level of IL-1β production in cell treated with rVvpM for 24 h was quantified by ELISA. *n* = 4. **(I)** Cells were incubated with rVvpM for 24 h. Percentages of necrosis, survival, and apoptosis were measured by using PI/Annexin V staining and flow cytometry (left panel). PI/Annexin V cells (Q1) were considered necrotic, PI/Annexin V double-positive cells (Q2) were considered late apoptotic, PI/Annexin V cells (Q3) were considered alive, and were PI/Annexin V cells (Q4) were considered early apoptotic. Quantitative analysis of the fold changes of necrotic (Q1) and apoptotic (Q2+Q4) cells by FACS analysis is shown (right panel). *n* = 3. ^*^P < 0.01 vs. Veh (boiled rVvpM). **(A–D)** ^*^P < 0.001 vs. Veh (boiled WT). ^#^*P* < 0.01 and ^##^*P* < 0.001 vs. WT, respectively. **(E–H)** ^*^P < 0.01 and ^**^P < 0.001 vs. cells with no treatment, respectively. ROD, relative optical density.

*V. vulnificus* (WT) secretes functional virulence factors, such as multifunctional autoprocessing repeats in the toxin (RTX) (Lee et al., 2007; Jeong and Satchell, 2012), elastase (VvpE) (Lee et al., 2015a,b, 2016), cytolytic pore-forming hemolysin (VvhA) (Jeong and Satchell, 2012; Song et al., 2016), and zinc-metalloprotease (VvpM) (Lee M. A. et al., 2014; Lee et al., 2015a). RAW 264.7 murine macrophage cells were exposed to *V. vulnificus* (WT) and various mutants deficient in *RTX, VvpE, VvhA*, and *VvpM* genes. A robust increase in the *IL*-*1*β mRNA expression level were observed 3 h after incubation with 1 MOI of WT (Figure 1A). WT also stimulated the expression of both pro-IL-1β and cleaved IL-1β (Figure 1B), and the secretion of IL-1β into the cell media (Figure 1C). Interestingly, LPS as a positive control has similar ability with WT in the induction of IL-1β expression and the release. However, the level of IL-1β was far less evident in cells infected with the *VvpM* mutant as compared to cells infected with other WT mutants. Among the various pro-inflammatory cytokines, the expression of *IL-1*β appeared to be highly susceptible to *VvpM* mutant infection (Figure 1D). To determine the direct effect of VvpM, cells were exposed to various concentrations (0~200 pg/mL) of the recombinant protein (r) VvpM purified by *V. vulnificus* for 24 h. A significant increase in *IL-1*β expression was observed after 3 h of incubation with 100 pg/mL of rVvpM (Figure 1E). rVvpM preferentially increased the level of *IL-1*β mRNA among the mRNA amplicons of pro-inflammatory cytokines (Figure 1F). Interestingly, an increase in the active form of IL-1β (17 kDa) for 12 h (Figure 1G) and its secretion into the cell media for 24 h (Figure 1H) were also observed in a time-dependent manner after treatment with rVvpM in both RAW 264.7 cells and Caco-2 human intestinal epithelial cells. We determined the functional role of rVvpM to induce cell death by means of flow cytometric analyses over a period of 24 h (Figure 1I). rVvpM significantly induced the necrosis (an 8.5 ± 0.3-fold increase compared to a vehicle) of the cells, whereas for apoptotic cell death, a marginal effect was noted (a 1.3 ± 0.2-fold increase compared to the vehicle). We further determined the functional role of rVvpM to induce cell death by staining with annexin V for apoptotic cells, PI for necrotic cells, and CytoCalcein for live cells. Consistent with our data for flow cytometric analyses, we found that rVvpM is essential for triggering the necrotic cell death rather than the apoptosis (Figure S2). We further confirmed the necrosis-promoting effect of rVvpM using another reagent that monitors necrotic cells with 7-aminoactinomycin D. As shown in Figure S3A, we found that rVvpM shows greater stimulatory potency on necrotic cell death than on apoptotic cell death, confirming that rVvpM is essential for triggering necrotic macrophage death rather than apoptosis. In addition, the levels of IL-1β and necrotic cell death remained unchanged after treatments with identical amounts of trypsin, boiled rVvpM, or boiled WT for 12 h (Figure S3B), suggesting that the functional roles of rVvpM during the processes of IL-1β production and necrotic cell death differ from the outcome of the general proteolysis of surface proteins or contamination by the bacterial product. Thus, these results indicate that rVvpM is responsible for triggering necrotic macrophage death during *V. vulnificus* infection and that it evokes a pro-inflammatory response via mainly IL-1β production.

### VvpM facilitates the lipid raft-mediated clustering of ANXA2

**Figure 2:**
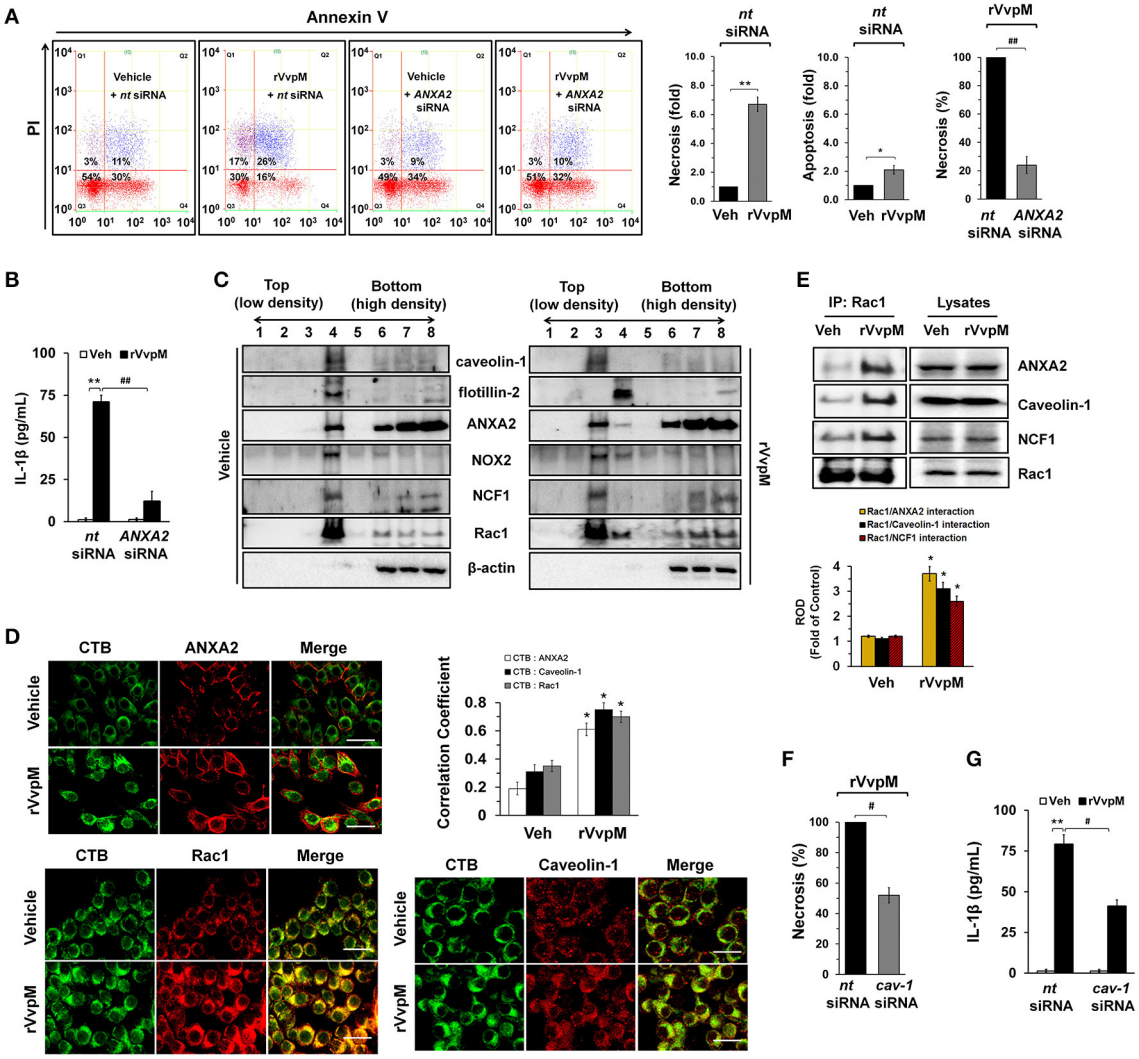
VvpM facilitates the lipid raft-mediated clustering of ANXA2. **(A)** Cells transfected with siRNAs for non-targeting (*nt*) control and ANXA2 were incubated with rVvpM for 24 h. FACS analysis and quantitative analysis of the percentage of necrotic (Q1) cells are shown. Data represent the means ± S.E. (*n* = 4). **(B)** The level of IL-1β protein was quantified by ELISA. *n* = 5. **(C)** Caveolin-enriched membrane fractions were prepared by discontinuous sucrose density gradient fractionation from the cell treated with rVvpM for 30 min, and the location of caveolin-1, flotillin-2, ANXA2, NOX2, NCF1, and Rac1 was determined by Western blot. *n* = 3. **(D)** The increased co-localization of CTB (green) with ANXA2 (red), caveolin-1 (red), and Rac1 (red) was determined by confocal microscopy using immunofluorescence staining. Scale bars, 100 μm (magnification, ×400). *n* = 3. Graphs represent Pearson's coefficient of colocalization of CTB and ANXA2, CTB and Caveolin-1, and CTB and Rac1 from 10 independent fields. ^*^P < 0.05. **(E)** Rac1 co-immunoprecipitated with ANXA2, caveolin-1, and NCF1 is shown in the left side. Expression of ANXA2, caveolin-1, NCF1 and Rac1 in total cell lysates is shown in the right side. *n* = 4. ^*^P < 0.01 vs. Veh (boiled rVvpM). ROD, relative optical density. **(F)** Cells transfected with *nt* siRNA and *cav-1* siRNA were incubated with rVvpM for 24 h. FACS analysis and quantitative analysis of the percentage of necrotic (Q1) cells are shown; *n* = 4. **(G)** The level of IL-1β protein was quantified by ELISA. *n* = 5. **(A,B,F,G)** ^*^P < 0.01 and ^**^P < 0.001 vs. *nt* siRNA + Veh (boiled rVvpM), respectively. ^#^*P* < 0.05 and ^##^*P* < 0.01 vs. *nt* siRNA + rVvpM, respectively.

It is well established that annexin A2 (ANXA2) plays a critical role as a host mediator of bacterial pathogens and is essential in the pathogenesis of *Enteropathogenic E. coli* infections (Zobiack et al., 2002). We have previously reported that exotoxins produced by *V. vulnificus* contributes to their pathogenesis in multiple ways by interacting with intestinal proteins, including ANXA2 and ANXA2. However, our previous data have revealed that ANXA2, but not ANXA4 plays a critical role as a membrane mediator of *V. vulnificus* and that it has unique biological properties responsible for host cell death coupled with an inflammatory response (Lee et al., 2015a). We initially determined the existence of ANXA isotypes in RAW264.7 cells. ANXAs are expressed in the order of *ANXA2, ANXA3, ANXA5*>*ANXA1, ANXA4, ANXA6* (Figure S4). The expressions of *ANXA7-13* were not detected in these cell lines. Interestingly, the knockdown of *ANXA2* resulted in a reduction of necrotic cell death (Figure 2A) and IL-1β production (Figure 2B), as elicited by rVvpM. In attempting to define the membrane location of functional ANXA2, our data revealed that the lipid raft markers caveolin-1 and flotillin-2 were found in mainly fraction 4, whereas ANXA2 was highly enriched both in the lipid raft parts (fraction 4) and the non-lipid raft parts (fractions 6–8) (Figure 2C). Moreover, the subunits of the NADPH oxidase (NOX) enzymes, NOX2 (gp91^phox^) and NCF1 (p47^phox^), and a cytosolic component of the NOX family, Rac1, were enriched in fraction 4. Given that caveolin-1 is constitutively expressed in lipid rafts to maintain cell homeostasis forming caveolin-1-mediated caveoli (Li et al., 2007), it is possible that individual lipid rafts attached with caveolin-1 are present in fraction 4. These individual lipid rafts were known to be dynamic microdomains and carry several membrane-bound or attached proteins or enzymes such as G-proteins, protein kinases, and NOX enzymes (Li et al., 2007). Interestingly, an increase in the level of ANXA2 together with caveolin-1, but not flotillin-2, appeared in fraction 3 after incubation with 100 pg/mL of rVvpM compared to the vehicle alone, suggesting that rVvpM enhances the recruitment of ANXA2 into a caveolin-1-enriched component. In addition, a treatment with rVvpM resulted in the translocations of NOX2, NCF1, and Rac1 into fraction 3 including the caveolin-1-enriched component. This indicate that rVvpM stimulates the movement and clustering of individual lipid rafts without flotillin-1 to form a number of lipid rafts macrodomains or platforms with aggregation or recruitment of receptors, NOX enzymes, and other proteins such as Rac1 at fraction 3. Regarding the exception of flotillin-1 move in in the cells activated by rVvpM, it reported that flotillin-1 is independently regulated and does not strictly correlate with the expression patterns of caveolin family members (Volonte et al., 1999). Indeed, it reported that the distribution patterns of flotillin 1 did not change upon cholesterol removal (Kaakinen et al., 2012). Therefore, our result in the present study indicate that rVvpM initiates the organization of rafts and the spatial distribution of caveolin which are crucially dependent on lipid raft clustering in fraction 3. The effect of rVvpM on the membrane location of ANXA2 was further visualized by staining the ANXA2 and the lipid raft marker molecule cholera toxin subunit B (CTB). As shown in Figure 2D, rVvpM significantly increased the co-localization of CTB with ANXA2. In addition to ANXA2, CTB significantly co-localized with caveolin-1 and Rac1 in the cells treated with rVvpM. Because the above approaches are qualitative at best, we also attempted to quantify the results by means of the co-immunoprecipitation of Rac1 with proteins related to the lipid rafts in the presence of rVvpM. It was noted that Rac1 co-immunoprecipitated with ANXA2, caveolin-1, and NCF1, and importantly, that these interactions were enhanced by the rVvpM treatment (Figure 2E). In good agreement with the above results, the knockdown of caveolin-1 significantly abrogated rVvpM-induced necrosis (Figure 2F) and the production of IL-1β (Figure 2G). These findings indicate that rVvpM facilitates the lipid-raft-mediated clustering of ANXA2 and NOX enzymes to trigger IL-1β production coupled with necrotic macrophage death.

### VvpM induces ROS production and PKCα phosphorylation

**Figure 3:**
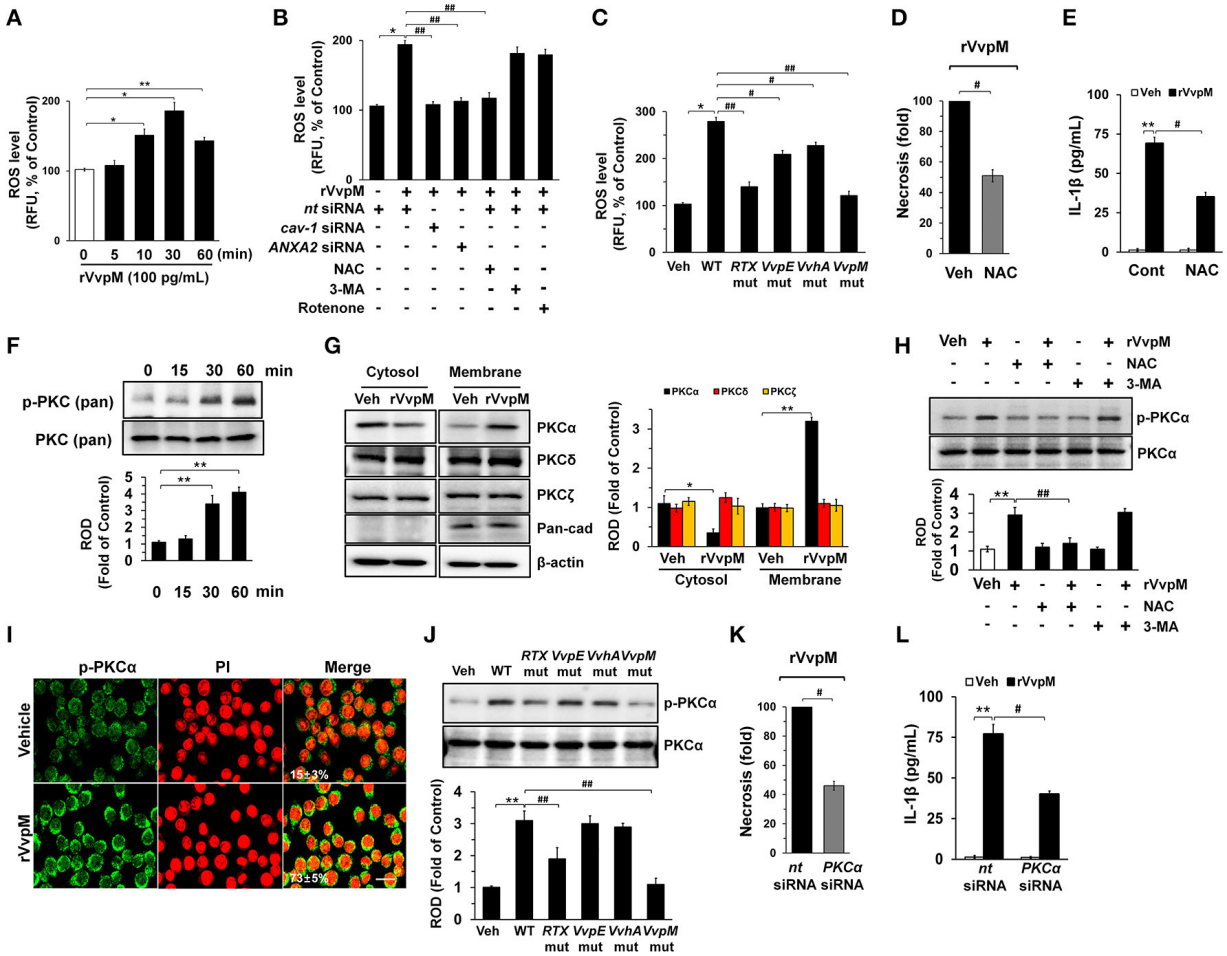
VvpM induces ROS production and PKCα phosphorylation. **(A)** Time responses of rVvpM in ROS production is shown. *n* = 4. **(B)** Cells were transfected with siRNAs for *cav-1* and *ANXA2* for 24 h or pre-treated with NAC (10 μM), 3-MA (10 mM), and rotenone (0.1 μM) for 30 min prior to rVvpM exposure for 30 min. *n* = 5. **(C)** Cells infected with WT or various mutant deficients for 30 min is shown. *n* = 4. **(D)** Cell were pre-treated with NAC (10 μM) for 30 min prior to rVvpM exposure for 24 h. FACS analysis and quantitative analysis of the percentage of necrotic (Q1) cells are shown. *n* = 4. **(E)** The level of IL-1β protein was quantified by ELISA. *n* = 5. **(F)** Phosphorylation of pan-PKC is shown. *n* = 3. **(G)** Membrane translocation of PKC isoforms in cells treated with rVvpM for 30 min is shown. Pan-cadherin was used as a membrane control. *n* = 3. **(H)** Phosphorylation of PKCα in cells pre-treated with NAC and 3-MA for 30 min prior to rVvpM exposure for 30 min is shown. *n* = 3. **(I)** Expression of PKCα (green) was determined by confocal microscopy. The cell numbers showing membrane translocalization of PKCα per microscopic filed were directly counted and converted to a percentage by multiplying by 100. Ten random fields per coverslip were counted. Data represent the mean ± S.E. *n* = 3. **(J)** Phosphorylation of PKCα in a cells infected with WT or various mutant deficients for 30 min is shown. *n* = 4. **(K)** Cells transfected with *PKC*α siRNA were incubated with rVvpM for 24 h. FACS analysis and quantitative analysis of the percentage of necrotic (Q1) cells are shown. *n* = 4. **(G)** The level of IL-1β protein was quantified by ELISA. *n* = 5. **(A,F)** ^*^P < 0.01 and ^**^P < 0.001 vs. cells with no treatment, respectively. **(B,K,L)** ^*^P < 0.05 and ^**^P < 0.01 vs. Veh (boiled WT), respectively. ^#^*P* < 0.05 and ^##^*P* < 0.01 vs. *nt* siRNA + rVvpM, respectively. **(C,J)** ^*^P < 0.05 and ^**^P < 0.01 vs. Veh (boiled WT), respectively. ^#^*P* < 0.05 and ^##^*P* < 0.01 vs. WT, respectively. **(D,E)** ^**^P < 0.01 vs. Veh (boiled rVvpM). ^#^*P* < 0.05 and ^##^*P* < 0.01 vs. Veh + rVvpM, respectively. **(G,H)** ^*^P < 0.01 and ^**^P < 0.001 vs. Veh (boiled rVvpM), respectively. ^##^*P* < 0.05 vs. rVvpM alone. ROD, relative optical density; RFU, relative fluorescence units.

We subsequently determined whether rVvpM regulates the production of ROS with regard to the functional role of the lipid raft-mediated clustering of NOX enzymes. We found that rVvpM significantly increased ROS production between 10 and 60 min after incubation with 100 pg/mL of rVvpM compared to the vehicle alone (Figure 3A). The ROS production induced by rVvpM at 30 min was significantly inhibited by the knockdown of *caveolin-1* and *ANXA2* and by a treatment with the positive control N-acetylcysteine (NAC) as an antioxidant (Figure 3B). Despite the significant role of rVvpM in promoting autophagy induction (Lee M. A. et al., 2015), the autophagy inhibitor 3-methyladenine (3-MA) did not affect rVvpM-induced ROS production, suggesting that the signaling events related to ROS production are unrelated to the characteristics of autophagy. Moreover, we found that mitochondrial complex I inhibitor, rotenone (0.1 μM) has a very weak inhibitory effect on ROS production induced by rVvpM for 30 min. Consistently, rVvpM did not show any significant effect on mitochondiral ROS production (Figure S5). These results indicate that epithelial ROS generated by clustering of NADPH oxidase enzymes within lipid rafts facilitate the production of ROS, which results in a prominent amplification of the transmembrane signal at short time period. Importantly, a prominent increase in ROS production was also observed in cells infected with WT (Figure 3C). In contrast, while mutants deficient in *VvpM* and *RTX* significantly decreased the ability of WT to induce ROS production, macrophages infected with *VvpE* and *VvhA* mutants revealed only a modest defect. Moreover, the instances of necrotic cell death (Figure 3D) and IL-1β production (Figure 3E) induced by rVvpM were significantly blocked by a pre-treatment with NAC. These results indicate that ROS production is required for VvpM to promote IL-1β production coupled with necrotic macrophage death. ROS produced by the clustering of NADPH oxidase enzymes results in a prominent amplification of the transmembrane signal through the activation of PKC (Wu et al., 2008; Zhang et al., 2010). rVvpM significantly induced pan-PKC phosphorylation between 30 and 60 min (Figure 3F). In an experiment to identify the specific PKC isotypes, the translocation of PKCα, but not PKCδ or PKCζ, from the cytosol to the membrane compartment was observed after cells were treated with rVvpM for 30 min (Figure 3G). Importantly, the increase in PKCα phosphorylation was significantly blocked by a pretreatment with a ROS scavenger, NAC, but not by 3-MA (Figure 3H), indicating that PKCα phosphorylation is a downstream factor of ROS production induced by rVvpM acting on the membrane lipid raft, which is not affected by autophagy. The membrane expression of PKCα phosphorylation was further confirmed by immunofluorescence staining in rVvpM-treated RAW264.7 cells (Figure 3I). Not surprisingly, while WT significantly increases the level of PKCα phosphorylation, the *VvpM* mutant showed the most similar level to that noted in control mice compared to the other mutant forms (Figure 3J), indicating that PKCα phosphorylation mediated by ROS is a more efficient infectious mechanism of VvpM as compared to RTX. It was noted that rVvpM induces phosphorylation of PKCα at Ser 657 site responsible for PKCα autophosphorylation that leads to stabilization and maturation of the PKCα. In addition, rVvpM significantly stimulated calcium influx, which was enhanced by A23187 as a positive control (Figure S6A). Interestingly, the silencing of PKCα by *PKC*α siRNA significantly blocked the necrotic cell death (Figure 3K) and IL-1β production (Figure 3L) induced by rVvpM. Given that previous result showing the PKC activation also able to stimulate the ROS production via phosphorylation of NOX subunits (Cosentino-Gomes et al., 2012), we have further examined whether PKCα regulates ROS production in RAW264.7 cells treated with rVvpM. However, the knockdown of PKCα failed to regulate ROS production induced by rVvpM (Figure S6B), PKCα is downstream factor of ROS during necrotic macrophage death induced by rVvpM. Together, these results indicate that rVvpM coupling with ROS production uniquely stimulates atypical PKC activation to promote IL-1β production and necrotic macrophage death.

### VvpM induces the activation of JNK and NF-κBp65 to promote IL-1β expression

**Figure 4:**
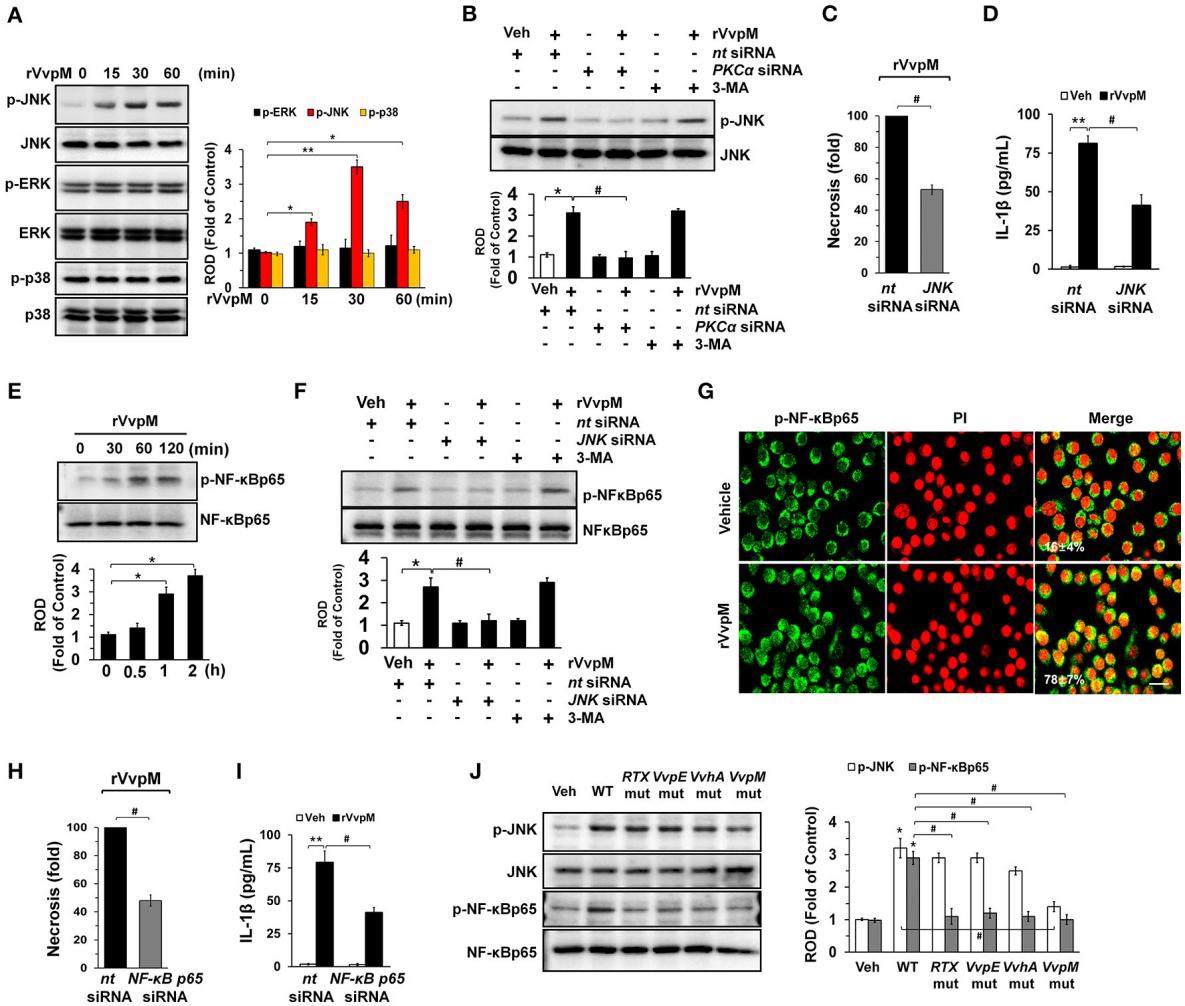
VvpM induces the activation of JNK and NF-κBp65 to promote IL-1β expression. **(A)** The effect of rVvpM on the expression of MAPK was determined by western blot. *n* = 3. **(B)** Cells were transfected with *PKC*α siRNA for 24 h prior to rVvpM exposure for 30 min. *n* = 3. **(C)** Cells were transfected with *JNK* siRNA for 24 h prior to rVvpM exposure for 24 h. FACS analysis and quantitative analysis of the percentage of necrotic (Q1) cells are shown. *n* = 4. **(D)** The level of IL-1β protein was quantified by ELISA. *n* = 5. **(E)** Time responses of rVvpM in the phosphorylation of NF-κBp65 are shown. *n* = 3. **(F)** Cells were transfected with *JNK* siRNA for 24 h or pre-treated with 3-MA (10 mM) for 30 min prior to rVvpM exposure for 60 min. *n* = 3. **(G)** p-NF-κBp65 (green) was detected by confocal microscopy. The cell numbers showing nuclear translocalization of p-NF-κBp65 per microscopic filed were directly counted and converted to a percentage by multiplying by 100. Ten random fields per coverslip were counted. Data represent the mean ± S.E. Scale bars, 100 μm. *n* = 3. **(H)** Cells transfected with *NF*-κ*Bp65*siRNA were incubated with rVvpM for 24 h. FACS analysis and quantitative analysis of the percentage of necrotic (Q1) cells are shown. *n* = 4. **(I)** The level of IL-1β protein was quantified by ELISA. *n* = 5. **(J)** Phosphorylation of JNK and NF-κBp65 in a cells infected with WT or various mutant deficients for 30 min are shown. *n* = 4. **(A,E)** ^*^P < 0.05 and ^**^P < 0.01 vs. cells with no treatment, respectively. **(B–D,F,H,I)** ^*^P < 0.01 and ^**^P < 0.001 vs. *nt* siRNA + Veh (boiled rVvpM), respectively. ^#^*P* < 0.01 vs. *nt* siRNA + rVvpM. **(J)** ^*^P < 0.01 vs. Veh (boiled WT). ^#^*P* < 0.01 vs. WT, ROD; relative optical density.

Many bacterial stimuli regulate the MAPKs and NF-κB pathways, which are interesting candidates as downstream mediators of ROS and PKC. rVvpM increased the phosphorylation of JNK between 15 and 60 min but does not affect the phosphorylation of ERK or p38 MAPK (Figure 4A), and its effect at 30 min is inhibited by the silencing of PKCα, but not by 3-MA (Figure 4B). In addition, the silencing of *JNK* by siRNA has a significant inhibitory effect on the necrosis (Figure 4C) and production of IL-1β (Figure 4D) in RAW264.7 cells. rVvpM also induced NF-κBp65 phosphorylation between 60 and 120 min (Figure 4E). Treatment with *JNK* siRNA but not 3-MA significantly blocked rVvpM-induced NF-κBp65 activation (Figure 4F). The increased accumulation of NF-κBp65 phosphorylation in the nucleus was further confirmed by immunofluorescence staining and counter-labeling with propidium iodide (PI) (Figure 4G). In agreement with this, the necrosis (Figure 4H) and production of IL-1β (Figure 4I) induced by rVvpM were significantly blocked by the knockdown of NF-κBp65. The effect of rVvpM on the activation of JNK and NF-κBp65 was further confirmed in these cells following infection with WT and various deficiency mutants (Figure 4J). WT significantly induced the activation of JNK and NF-κBp65, whereas for the *VvpM* mutant, only a marginal effect was noted, and the cells appeared to be highly susceptible to JNK among the various mutants tested here, although all four deficiency mutants caused significant defects in the ability of WT to activate NF-κBp65. Consistently, our results have revealed that all mutations have inhibitory effects on transcription activity of NF-κB induced by WT in RAW 264.7 and Caco-2 cells (Figures S7A,B). The data above provide important evidence that JNK-mediated NF-κBp65 phosphorylation is required for the regulation of IL-1β production and necrotic cell death as evoked by rVvpM. In addition, the phosphorylation of JNK and NF-κB induced by rVvpM was significantly inhibited by a treatment with a ROS scavenger N-acetylcysteine (NAC) (Figure S7C), suggesting that ROS induced by rVvpM plays key role in activation of PKC, JNK, and NF-κB in the promotion of necrotic macrophage death

**Figure 5:**
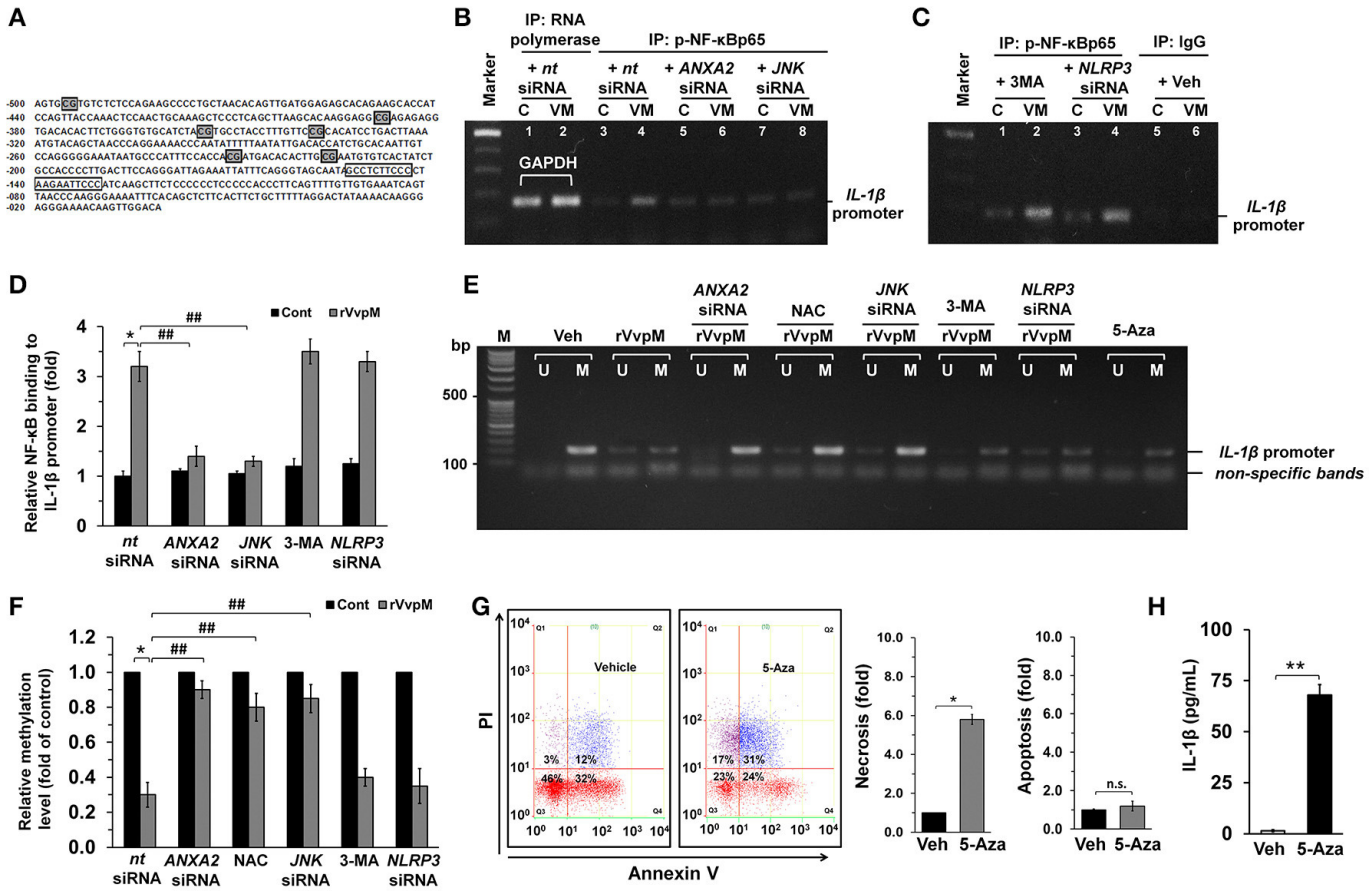
Regulatory effect of VvpM on the interaction of NF-κB with the *IL-1*β promoter. **(A)** The NF-κB binding sites and methylation site in the *IL-1*β promoter are shown. The open boxes and shaded box represent the consensus sequences of NF-κB and the methylation site, respectively. **(B,C)** Cells were transfected with siRNAs for *ANXA2, JNK*, and *NLRP3* for 24 h or pre-treated with 3-MA (10 mM) for 30 min prior to rVvpM exposure for 60 min. Cells were then fixed with formaldehyde, and cell extracts were immunoprecipitated (IP) with anti-p-NF-κBp65 to detect bound *IL-1*β promoter DNA fragments. A representative agarose gel following RT-PCR is shown. *n* = 3. Normal mouse IgG or anti-RNA polymerase II antibody was used as negative or positive control for the ChIP, respectively. C, control. VM, rVvpM. **(D)** The level of p-NF-κBp65 binding to *IL-1*β promoter was quantified by qRT-PCR. Anti-RNA polymerase II antibody was used internal control. *n* = 3. **(E)** Genomic DNA of the cells transfected with siRNAs for ANXA2, JNK, and NLRP3 for 24 h or pre-treated with NAC (10 μM), 3-MA (10 mM), and 5-aza (1 μM) for 30 min prior to rVvpM exposure for 60 min was prepared. Changes in the *IL-1*β promoter methylation status were determined by MSP analysis. U, unmethylated form. M, methylated form. **(F)** The level of *IL-1*β methylation was quantified by qRT-PCR. Relative level of *IL-1*β methylation is shown, compared to the unmethylated form. *n* = 4. **(G)** Cell were treated with 5-aza (1 μM) for 24 h. FACS analysis (left panel) and quantitative analysis of the fold changes of necrotic (Q1) and apoptotic (Q2+Q4) cells by FACS analysis is shown (right panel). *n* = 4. **(H)** The level of IL-1β protein was quantified by ELISA. *n* = 5. **(D,F)** ^*^P < 0.01 vs. *nt* siRNA + Veh (boiled rVvpM). ^##^*P* < 0.01 vs. *nt* siRNA + rVvpM. **(G,H)** ^*^P < 0.01 and ^**^P < 0.001 vs. cells with no treatment, respectively.

To determine the role of NF-κB in the transcriptional regulation of *IL-1*β mRNA expression, we conducted chromatin immunoprecipitation (ChIP) assays followed by qRT-PCR in cells treated with rVvpM. With ALGGEN PROMO (Messeguer et al., 2002; Farre et al., 2003), we found that the *IL-1*β promoter located between the transcription start site and the point–500 bp upstream contains two putative NF-κB binding sites and six methylation sites. Test results also found a primer that includes two putative NF-κB binding sites at the region proximal −56 bp to −237 bp of the start site of the *IL-1*β promoter (Figure 5A). Figure 5B shows that our primer sets result in an amplicon from the anti-phospho-NF-κBp65 immunoprecipitates and, importantly, that the interaction between NF-κB and the *IL-1*β promoter was enhanced by a rVvpM treatment for 60 min. Interestingly, however, the level of NF-κBp65 binding to the *IL-1*β promoter was significantly inhibited by the silencing of ANXA2 and JNK (Figure 5B) but not by treatments with 3-MA or *NLRP3* siRNA, which is a critical regulator of IL-1β production (Lamkanfi et al., 2011; Guo et al., 2015; Figure 5C). The ability of rVvpM to induce interaction between NF-κB and the IL-1β promoter is consistent with the data from a real-time PCR analysis (Figure 5D). These results indicate that rVvpM acting on the lipid raft-mediated clustering of ANXA2 transcriptionally regulates the NF-κBp65 binding to the *IL-1*β promoter via JNK activation. Subsequently, we postulated that rVvpM may affect methylation, which negatively regulates the interaction between NF-κB and the *IL-1*β promoter. We attempted to analyze the methylation status of the *IL-1*β gene promoter containing the −328, −294, and −277 CpG sites due to their close proximity to putative NF-κB binding sites. As shown in Figure 5E, a primer pair including these CpG sites that specifically amplified either the methylated or unmethylated form of the *IL-1*β promoter produced 203 bp methylated bands from vehicle-treated RAW264.7 cells, indicating that the *IL-1*β promoter was methylated in these alleles. However, treatment with 100 pg/mL of rVvpM for 60 min markedly inhibited the level of *IL-1*β promoter methylation, which was significantly blocked by the knockdown of *ANXA2* and *JNK* and by a pre-treatment with NAC. As expected, treatment with 3-MA and *NLRP3* siRNA failed to regulate the methylation status of the *IL-1*β gene promoter induced by rVvpM. These results were consistent with the data from a real-time PCR analysis quantified by the relative value of CpG methylation compared to an unmethylated form in the *IL-1*β promoter (Figure 5F). Interestingly, the levels of necrotic cell death (Figure 5G) and the production of IL-1β (Figure 5H) were markedly enhanced by a pre-treatment with the DNA methylation inhibitor 5-azacytidine (5-aza). These results suggest that the hypomethylation of the *IL-1*β promoter is able to affect the high level of the interaction between NF-κB and the *IL-1*β promoter during necrotic macrophage death as evoked by rVvpM.

### VvpM induces autophagy by regulating non-lipid raft ANXA2

**Figure 6:**
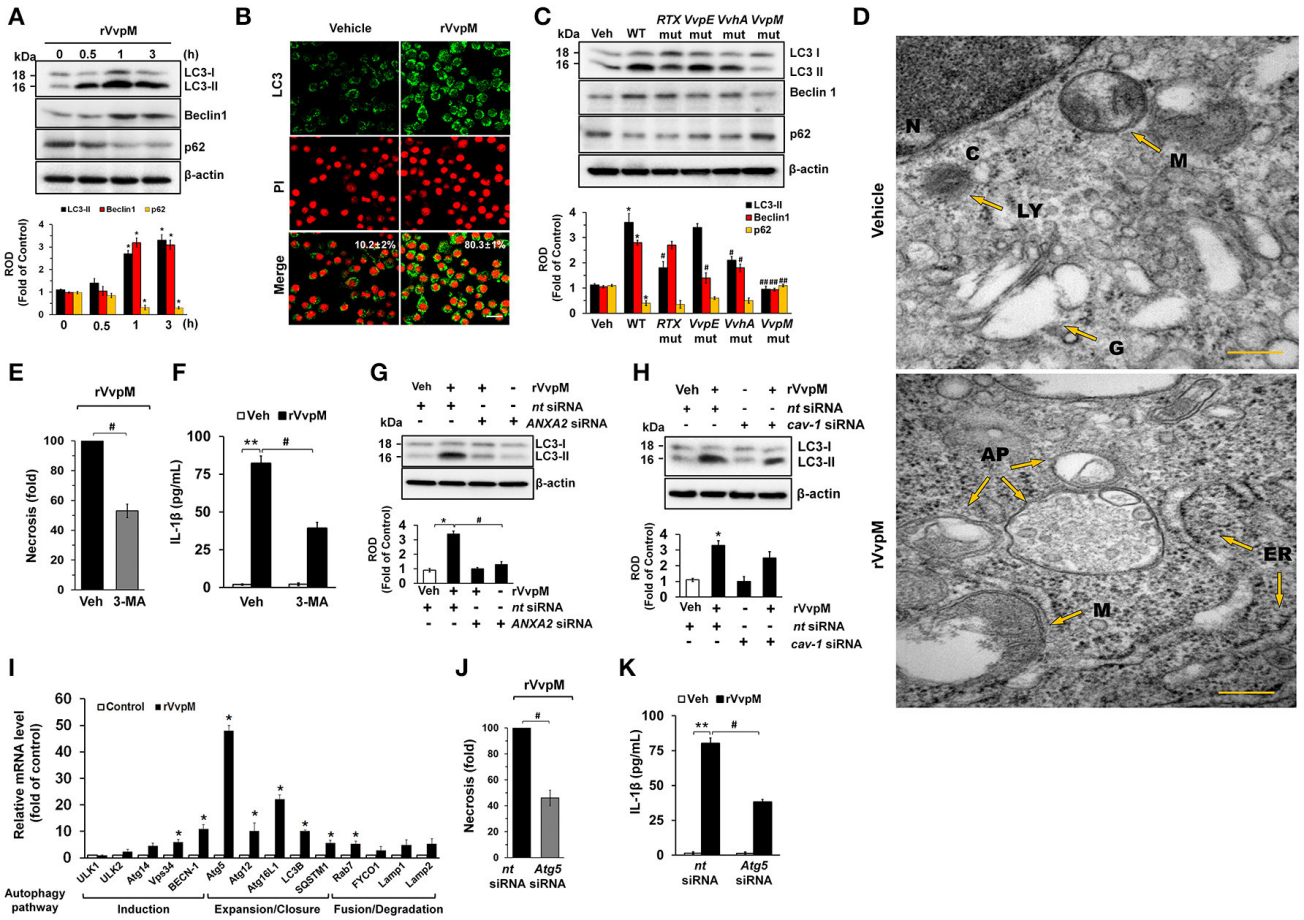
VvpM induces autophagy by regulating non-lipid raft ANXA2. **(A)** The effect of rVvpM on expressions of autophagy-related proteins, LC3, Beclin-1, and p62 were determined by Western blot. Data represent the mean ± S.E. *n* = 4. **(B)** LC3 expression (green) in cells treated with rVvpM for 3 h was visualized by confocal microscopy and quantified by using Image J program, which measures the stained area per microscopic filed with consistent threshold. Scale bars, 100 μm. *n* = 4. **(C)** The expressions of autophagy-related proteins in a cells infected with WT or various mutant deficients for 1 h are shown. *n* = 4. ^*^P < 0.05 vs. Veh (boiled WT). ^#^*P* < 0.05 and ^##^*P* < 0.01 vs. WT, respectively. **(D)** Formation of autophagic vesicles in cells treated with rVvpM for 3 h was visualized by transmission electron microscopy. Scale bars, 100 nm. *n* = 3. AP, autophagosome; C, cytosol; ER, endoplasmic reticulum; N, nucleus; G, golgi body; M, mitochondria. **(E)** Cells were pre-treated with 3-MA (10 mM) for 30 min prior to rVvpM exposure for 24 h. FACS analysis and quantitative analysis of the percentage of necrotic (Q1) cells are shown. *n* = 4. **(F)** The level of IL-1β protein was quantified by ELISA. *n* = 5. LC3 expression in cells transfected with siRNAs for *ANXA2* **(G)** and *cav-1* **(H)** for 24 h prior to rVvpM exposure for 3 h. **(I)** The effect of rVvpM on the expression of autophagy-related proteins was evaluated by qRT-PCR. The expression level of Atg5 and Atg16L1 was markedly increased by rVvpM treatment. *n* = 4. **(J)** Cells transfected with *Atg5* siRNA were incubated with rVvpM for 24 h. FACS analysis and quantitative analysis of the percentage of necrotic (Q1) cells are shown. *n* = 4. **(K)** The level of IL-1β protein was quantified by ELISA. *n* = 5. **(A,I)** ^*^P < 0.01 vs. cells with no treatment. **(E,F)** ^**^P < 0.01 vs. Veh (boiled rVvpM). ^#^*P* < 0.05 vs. Veh + rVvpM. **(G,H,J,K)** ^*^P < 0.05 and ^**^P < 0.01 vs. *nt* siRNA + Veh (boiled rVvpM), respectively. ^#^*P* < 0.05 and ^##^*P* < 0.01 vs. *nt* siRNA + rVvpM, respectively. ROD, relative optical density.

Despite the independent roles of autophagy and inflammasome in regulating the ROS-PKCα-JNK-NF-κBp65 pathway through the lipid-raft-mediated clustering of ANXA2, we revisited the issue of whether rVvpM has the ability to regulate the autophagic process and inflammasome formation due to the fundamental parts of these processes play role in regulating the production of IL-1β during bacterial infection. Importantly, while the expressions of LC3-II and Beclin-1 were increased by a treatment with rVvpM in a time-dependent manner, the level of p62 was attenuated (Figure 6A), indicating that rVvpM has the ability to regulate the autophagic process. We also confirmed through confocal microscopy that rVvpM increases autophagic vesicle formation, during which a significant increase in LC3 puncta positive cells was observed (Figure 6B). Comparing the functional roles of VvpM with those of other virulence factors which evoke the autophagic responses, we found that WT markedly induced the expression levels of LC3-II and Beclin-1 while inhibiting that of p62 (Figure 6C). In contrast, cells infected with the *VvpM* mutant among all cells infected with the mutants tested here exhibited the lowest LC3-II level similar to that of the control, suggesting that VvpM is a major virulence factor responsible for autophagy induction. Moreover, the functional effects of the *VvpM* mutant in LC3 activation were significantly restored by the complementation of the VvpM mutant with a functional VvpM gene (Figure S8). A unique feature of autophagosomes characterized by the peculiar double-membraned vesicle was also observed in cells treated with rVvpM by transmission electron microscopy (TEM), where rVvpM caused significant abnormalities such as swollen mitochondria while also widening the endoplasmic reticulum (ER) (Figure 6D). To confirm the enhanced autophagic flux, we used bafilomycin A1 (BafA1) which is a known inhibitor of V-ATPase (vacuolar-type H^+^ ATPase) that prevents the maturation of autophagic vacuoles. rVvpM significantly enhanced the accumulation of LC3-II in cells pretreated with BafA1 (Figure S9), suggesting that the lysosomal degradation pathway was not inhibited by rVvpM. Importantly, the autophagy inhibitor 3-MA significantly blocked necrotic cell death (Figure 6E) and IL-1β production (Figure 6F) as induced by rVvpM. This indicates that rVvpM differentially regulates IL-1β production through the induction of autophagic macrophage death. It was unclear as to whether the activation of two different signaling pathways for the regulation of IL-1β production reflects the selective interaction between VvpM and the lipid raft. We focused on the fact that ANXA2 was also highly enriched in the non-lipid raft parts of the cell membrane. Notably, the level of LC3-II evoked by rVvpM was markedly inhibited by the knockdown of ANXA2 with *ANXA2* siRNA (Figure 6G). However, the knockdown of caveolin-1 (Figure 6H) and treatment with the lipid raft sequester MβCD failed to regulate the level of LC3-II in cells treated with rVvpM (Figure S10). These results indicate the ANXA2 in the non-lipid raft parts is functionally relevant for autophagy induction and that it distinctively regulates necrotic macrophage death and the pro-inflammatory response as a host mediator of rVvpM. To gain insight into how ANXA2 influences the autophagic process via the non-lipid raft parts, we further determined the effect of rVvpM on the expression of mRNA for induction, expansion/closure, and fusion/degradation in an autophagy system (Figure 6I). The prominent expressions of *Atg5* and *Atg16L1* for autophagy expansion/closure were observed following an rVvpM treatment with a trend toward increased mRNA expressions in an overall autophagy system. Intriguingly, the knockdown of *Atg5* in RAW264.7 cells reduced the levels of necrotic cell death (Figure 6J) and IL-1β production (Figure 6K), suggesting the involvement of Atg5 in IL-1β production coupled with autophagic macrophage death elicited by rVvpM.

### VvpM facilitates the formation of NLRP3 inflammasomes via autophagy activation

**Figure 7:**
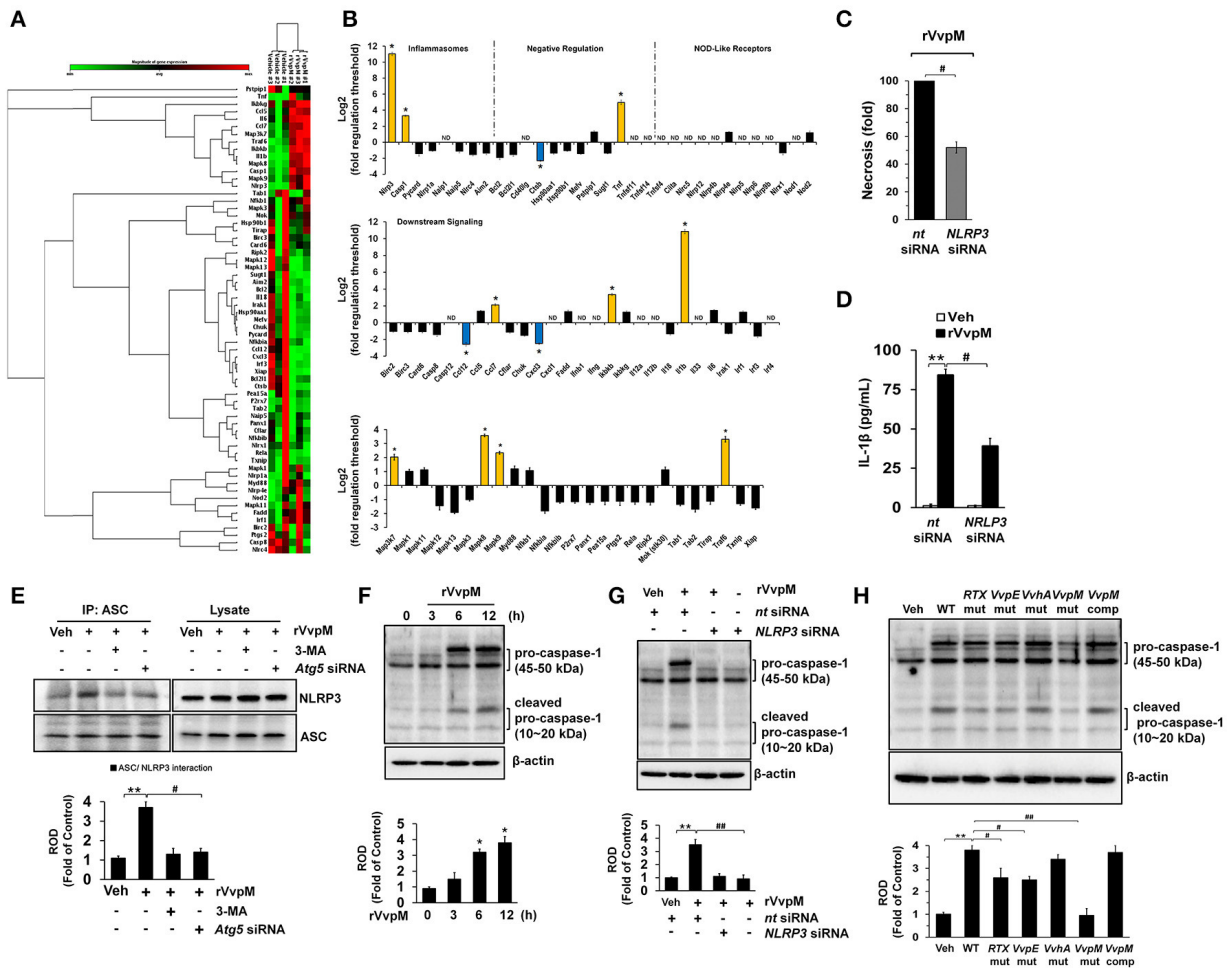
NLRP3 inflammasome is a downstream mediator of autophagy. **(A,B)** The mRNA expression levels of genes within the inflammasome pathway in cells treated with rVvpM for 24 h are shown. Expression of 84 genes involved in the inflammasome pathway assessed by RT^2^ Profiler PCR array, according to the manufacturer's instructions. Heat maps were generated and genes hierarchically clustered by Euclidean distance and single linkage by using the GeneGlobe Data Analysis Center on QIAGEN's website at http://www.qiagen.com/kr/shop/genes-and-pathways/data-analysis-center-overview-page/. *n* = 3. ^*^P < 0.01 vs. Veh (boiled rVvpM). **(C)** Cells transfected with *NLRP3* siRNA were incubated with rVvpM for 24 h. FACS analysis and quantitative analysis of the percentage of necrotic (Q1) cells are shown. *n* = 4. **(D)** The level of IL-1β protein was quantified by ELISA. *n* = 5. **(E)** ASC co-immunoprecipitated with NLRP3 is shown (left side). Expression of ASC and NLRP3 in total cell lysates is shown (right side). *n* = 4. ^**^P < 0.01 vs. Veh (boiled rVvpM). ^#^*P* < 0.05 vs. rVvpM alone. **(F)** The effect of rVvpM on the expression of caspase-1 was determined by western blot. *n* = 3. ^**^P < 0.01 vs. cells with no treatment. **(G)** Cells transfected with *NLRP3* siRNA were incubated with rVvpM for 6 h. *n* = 3. ^**^P < 0.01 vs. *nt* siRNA + Veh (boiled rVvpM). ^##^*P* < 0.01 vs. *nt* siRNA + rVvpM. **(H)** The expression of caspase-1 in a cells infected with WT, various mutant deficient, or complemented *VvpM* mutant for 2 h are shown. *n* = 4. ^**^P < 0.01 vs. Veh (boiled WT). ^#^*P* < 0.05 vs. WT. **(E–H)** ROD, relative optical density.

Given that *RTX* and *VvhA* produced by *V. vulnificus* are also known to cause the death of phagocytic cells via NLRP3-dependent caspase-1 activation *in vitro* and *in vivo* (Toma et al., 2010; Lo et al., 2011), it is important to clarify whether rVvpM regulates the expression of genes associated with the assembly, activation, and down-stream signaling of inflammasomes, which are linked to autophagy (Yuk and Jo, 2013). Our results from a RT^2^ Profiler PCR array showed that expression changes were detected in 13 of the 84 genes studied here after a 24 h rVvpM treatment (Figures 7A,B). Expression levels were increased in 10 genes (>two-fold, as shown by the green bar graph) but decreased in the other three genes (>two-fold, as shown by the blue bar graph). The remaining 61 genes showed no significant change in comparison with a vehicle treatment. The 10 genes showing increased expression levels were *NLRP3* (NM_145827), *Casp1* (caspase-1, NM_009807), *Tnf* (tumor necrosis factor, NM_013693), *Ccl7* (chemokine ligand 7, NM_013654), *Ikbkb* (IKK-β, NM_010546), *IL*-*1*β, *Map3k7* (TGF-β activated kinase 1, NM_172688), *Mapk8* (JNK1, NM_016700), *Mapk9* (JNK2, NM_016961), and *Traf6* (Tnf receptor-associated factor 6, NM_009424), while those showing decreased expression levels were *Ctsb* (cathepsin B, NM_007798), *Ccl12* (chemokine ligand 12, NM_011331), and *Cxcl3* (chemokine (C-X-C motif) ligand 3, NM_203320). Among these differentially regulated genes, we identified two genes (*IL*-*1*β and *NLRP3*) which were highly expressed in cells treated with rVvpM compared to a treatment with a vehicle (>10-fold). Importantly, the necrotic cell death (Figure 7C) as well as the production of IL-1β (Figure 7D) induced by rVvpM were significantly abrogated by NLRP3 knockdown. These results indicate VvpM is another virulence factor of *V. vulnificus* to regulate NLRP3 inflammasome responsible for IL-1β production coupled with necrotic macrophage death. Consistently, our results have revealed that rVvpM has ability to induce speck formation of pyrin domain of the adaptor protein, ASC (apoptosis-associated speck-like protein containing a CARD) (Figure S11). We also found that the ASC was co-immunoprecipitated with NLRP3, and importantly, that the interaction between ASC and NLRP3 was enhanced by a treatment with rVvpM (Figure 7E). In contrast, these interactions induced by rVvpM were significantly blocked by a treatment with the autophagy inhibitor 3-MA and knockdown of *Atg5* expression, suggesting that the formation of NLRP3 inflammasomes induced by rVvpM is an autophagy-dependent process. An increase in the active form of caspase-1 was also observed after 6 h of incubation with 100 pg/mL of rVvpM (Figure 7F). However, the activation of caspase-1 induced by rVvpM was markedly inhibited by transfection with siRNAs for *NLRP3* (Figure 7G). Importantly, WT significantly induced the activation of caspase-1 and IL-1β maturation, whereas the *VvpM* mutant showed a marginal effect and appeared to be highly susceptible among the various mutants (Figure 7H). Overall, these novel findings demonstrate that VvpM facilitates the formation of NLRP3 inflammasomes via autophagy activation to regulate caspase-1-mediated IL-1β production.

### VvpM contributes to the intestinal colonization of *V. vulnificus*

**Figure 8:**
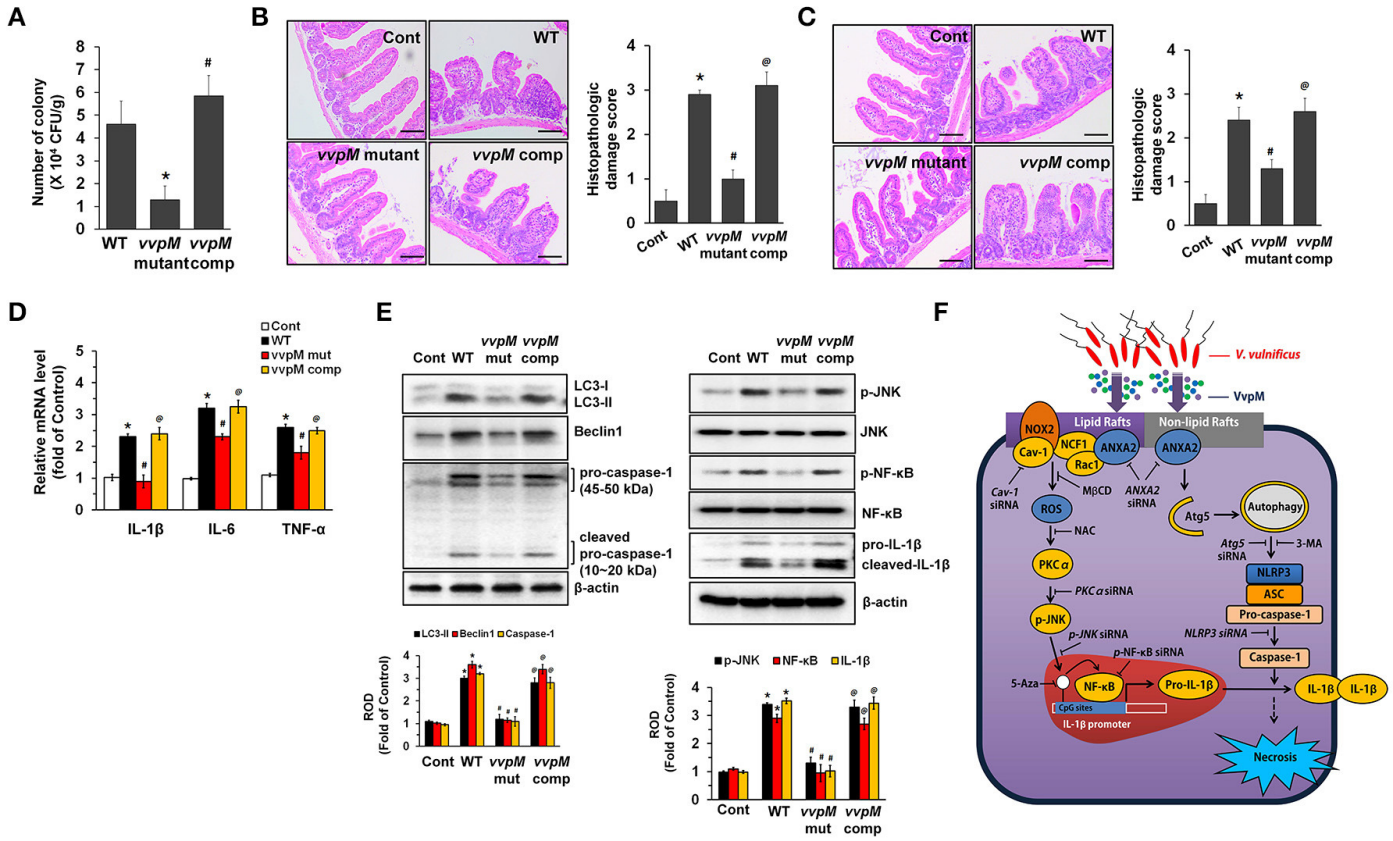
VvpM contributes to the intestinal colonization of *V. vulnificus*. **(A)** Mice inoculated with WT, boiled WT (Cont), VvpM mutant, and VvpM complement (comp) at 1.3 × 10^9^ CFU/mL, and sacrificed 16 h later. Colonization activities were determined. *n* = 10. **(B)** Representative ileum tissues stained with H&E are shown (left panel). *n* = 10. Scale bars represent 100 μm. Average scores of histopathologic damage index from mouse ileum is shown (right panel). *n* = 10. ^*^P < 0.01 vs. Cont. ^#^*P* < 0.05 vs. WT. ^@^*P* < 0.05 vs. *VvpM* mutant. **(C)** Closed ileal loop in mice was instilled with 100 μl of PBS containing WT, boiled WT (Cont), VvpM mutant, or *VvpM* complement (comp) at 1.3 × 10^9^ CFU/mL, and sacrificed 2 h later. Representative ileum tissues stained with H&E are shown (left panel). *n* = 10. Scale bars represent 100 μm. Average scores of histopathologic damage index from mouse ileum is shown (right panel). *n* = 10. ^*^P < 0.05 vs. Cont. ^#^*P* < 0.05 vs. WT. ^@^*P* < 0.05 vs. *VvpM* mutant. **(D)** The expression levels of pro-inflammatory cytokines (IL-1β, IL-6, and TNF-α) in ileal-ligated mouse model are shown. *n* = 5. ^*^P < 0.01 vs. Cont. ^#^*P* < 0.05 vs. WT. ^@^*P* < 0.05 vs. *VvpM* mutant. **(E)** The expression of LC3-II, Beclin 1, Caspase-1 and IL-1β, and the phosphorylation of JNK and NF-κB were determined by western blot. *n* = 4. ^*^P < 0.01 vs. Cont. ^#^*P* < 0.01 vs. WT. ^@^*P* < 0.01 vs. *VvpM* mutant. **(F)** A hypothetical model for VvpM-induced signaling pathway, where VvpM regulates two pathogenic pathways that stimulate NF-κB-dependent IL-1β production and autophagy-mediated NLRP3 inflammasome via spatial targeting distinct ANXA2.

To determine whether the functional role of VvpM in promoting IL-1β production coupled with macrophage cell death contributes to the growth of *V. vulnificus* in the gut, we further determined the level of bacterial colonization in mice inoculated intragastrically with a control, WT, and with the VvpM mutant and VvpM complementation (comp) at 1.3 × 10^9^ CFU/mL for 16 h (Figure 8A). WT colonization in the ileum was increased by 4.6 ± 1.1 (× 10^6^ CFU/g tissue) at 16 h. However, when mice were inoculated with the *VvpM* mutant, the levels were diminished by 3.3 ± 0.6 (× 10^6^ CFU/g tissue) compared to those associated with the WT. In contrast, complementation of the *VvpM* mutant with a functional *VvpM* gene completely reversed the effect of WT on ileal colonization. It was noted that all of the mice given oral administrations of WT had survived at 18 h post-injection (data not shown). WT also induced severe inflammation of the intestine, where it caused shortened villi heights accompanied by increased numbers of inflammatory cells at 16 h infection, resulting in an increased histopathological damage score compared to that in the control mice (Figure 8B). However, the *VvpM* mutant significantly failed to regulate intestinal villi structures and the related inflammation. In contrast, complementation of the *VvpM* mutant with a functional *VvpM* gene restored the effect of the *VvpM* mutant on the histopathological damage score. These results indicate that VvpM contributes to *V. vulnificus* colonization, facilitating pro-inflammatory responses *in vivo*.

To eliminate the possibility that the lower inflammatory responses evoked by the *VvpM* mutant can simply be explained by the presence of less bacteria, we undertook an additional experiment using the ileal-ligated mouse model in which a closed ileal loop was instilled with 100 μl of PBS containing WT, the *VvpM* mutant, or *VvpM* complementation at 1.3 × 10^9^ CFU/mL for 2 h. We anticipated that 2 h was the minimum duration for enterotoxicity and pro-inflammatory responses based on our recent report (Kim et al., 2013). There were no statistical differences between the groups treated with WT and the *VvpM* mutant in terms of the bacterial growth (data not shown). However, the injection of mice with the *VvpM* mutant at a time of 2 h failed to elevate the histopathological damage score (Figure 8C) or the expression levels of pro-inflammatory cytokines (Figure 8D) as caused by WT infection. The results in Figure 8E revealed that inoculation with WT for 2 h significantly increased the expressions of LC3-II, Beclin 1, Caspase-1 and mature IL-1β as well as the phosphorylation of JNK and NF-κB in mice, which were prevented by infection with the *VvpM* mutant. Notably, the functional effects of the *VvpM* mutant were significantly restored by the complementation of the VvpM mutant with a functional VvpM gene (Figure 8E). Thus, these results suggest that VvpM is a major virulence factor of *V. vulnificus* responsible for IL-1β production coupled with necrotic cell death.

## Discussion

In the present study, we demonstrate that VvpM mainly targets the critical pathogenic pathway of *V. vulnificus* to promote IL-1β production coupled with necrotic macrophage death. A key finding of our study is that VvpM acting through ANXA2 organized into lipid rafts stimulates the ROS-mediated NF-κB pathway to result in the transcriptional induction of IL-1β, whereas ANXA2 organized into non-lipid rafts induces activation of autophagy and NLRP3 inflammasome, resulting in caspase-1-mediated IL-1β maturation and necrotic macrophage death. VvpM may stimulate IL-1β production via ANXA2, which would remain segregated from raft elements in non-lipid rafts of the macrophage, while a specific raft-associated ANXA2 in response to VvpM would additively promote IL-1β production by co-clustering with other proteins recruited to rafts to enhance their affinity for the distinct raft subtype caveolin-1. We suggest that this is one of the reasons why VvpM makes a greater contribution to IL-1β production than the contributions of all other subtypes of exotoxins produced by *V. vulnificus*. Hence, these findings demonstrate that ANXA2 activated by VvpM via an identical extracellular cue regulates two pathogenic pathways closely related to IL-1β production via its spatial placement within the cell. Annexins are a well-known multigene family of calcium- and phospholipid-binding protein (Gerke and Moss, 2002). Having shown that ANXA2 has an important role in inflammatory responses responsible for IL-1β production during *V. vulnificus* infection (Lee et al., 2015a, 2016), our current results indicate that ANXA2 is highly expressed in macrophages and that spatial targeting of ANXA2 is required for IL-1β production coupled with necrotic macrophage death. In contrast to our results, phenotypes observed in ANXA2 knockout mouse models revealed that ANXA2 functions as a mediator of the anti-inflammatory response to maintain host defense mechanisms (He et al., 2016). We believe that these opposing ANXA2 effects are, in part, due to the presence of multiple ANXA receptors that have compensatory and redundancy actions with each other, and that these functions may vary depending on cellular characteristics and stress conditions. In fact, the phenotypes of most knockout models of annexin family have also been reported to be rather subtle, and all mice strains lacking one or even two annexins are viable and develop normally (Grewal et al., 2016). Compelling evidence also supports the critical role of ANXA2 in inflammatory myopathies (Probst-Cousin et al., 2004) and in inflammatory bowel disease (Tsukamoto et al., 2013). Thus, we contend that ANXA2 functions are linked to its ability to mediate the pro-inflammatory response of macrophages during *V. vulnificus* infection. Given that ANXA2 is not a transmembrane protein, it is very likely unable to transmit the signal initiated by the binding of VvpM. Therefore, it is possible that ANXA2 is associated with additional proteins that transfer the signal generated by VvpM into the macrophage. Our results indicate that VvpM may utilize reversible and multiple functions of ANXA2 to transmit the signal into the cell as a unique infectious stratagem, although the exact mechanism of VvpM interactions with ANXA2 remains undescribed. Alternatively, ANXA2 may merely function as an anchor protein to facilitate the binding of VvpM to the cell surface. Indeed, the importance of ANXA2 during microbial pathogenesis is underscored by the finding that *EPEC* adherence induces the aggregation of lipid rafts at sites of bacterial contact, where ANXA2 is recruited to the cytoplasmic membrane surface, possibly stabilizing raft patches and their links to the actin cytoskeleton beneath adhering to EPEC (Zobiack et al., 2002). Thus, our results indicate the possibility that rVvpM influences the structure and localization of ANXA2 through lipid rafts to promote pro-inflammatory signaling in macrophages.

With regard to lipid raft signaling events, VvpM induces the transcription of the IL-1β gene through the ROS-PKCα-JNK-NF-κB pathway via ANXA2. NADPH oxidase is a critical source of cellular ROS and has been studied extensively in phagocytes as an innate host defense component (Quinn and Gauss, 2004; Li et al., 2007). Increasing evidence has suggested that lipid rafts are clustered to form a redox-signaling platform through activation of NOX2 by interacting with NCF1 and Rac1 for membrane ROS production (Kusumoto et al., 2005; Li et al., 2007). Moreover, multiple signaling processes, such as those acting through the Ca^2+^ and PKC/MAPK pathways, are rapidly activated in target cells through ROS production (Lee et al., 2009, 2010). In fact, *H. pylori* and *C. perfringens* have also been shown to elicit a Ca^2+^ response (Fasano, 2002). Moreover, ANXA2 is a Ca^2+^/lipid-binding protein that differs from most other Ca^2+^-binding proteins, and the binding of Ca^2+^ to ANXA2 has been shown to trigger exposure of its N-terminal region, making it available for additional interactions/activities (Rescher and Gerke, 2004). Many studies have suggested a critical role of conventional PKCα in the cell death process during *EPEC* or *C. perfringens* infections (Crane and Vezina, 2005; Monturiol-Gross et al., 2014). These previous results are consistent with our current result showing that rVvpM may induce an influx of Ca^2+^ upon activation of ANXA2 and PKCα, as required for ROS production to stimulate IL-1β production and necrotic macrophage death. Despite the frequent involvement of ERK and p38 MAPK in the ROS signaling pathway induced by *H. pylori* infection (Ki et al., 2008) and *V. vulnificus* VvhA (Lee et al., 2015c), our results reveal that VvpM uniquely regulates IL-1β production through activation of the JNK-mediated NF-κB pathway. These results imply a functional role of rVvpM in the determination of downstream targets. Moreover, earlier research has shown that the JNK pathway induced by ROS can influence NF-κB activation in promoting apoptotic cell death (Seo et al., 2013). Hence, it is conceivable that ROS induced by rVvpM has a potential role in promoting the NF-κB pathway through activation of PKCα/JNK cascades. While the NF-κB binding activity of the *IL-1*β gene promoter is a critical mechanism in the transcriptional regulation of *IL-1*β, the promoter methylation of cytosine residues at CpG dinucleotides is also an important post-translational modification affecting NF-κB transcription. Indeed, it was clearly shown that ROS induced by H_2_O_2_ treatment regulates histone acetyltransferases (HAT) and deacetylases (HDAC), key enzymes responsible for chromatin remodeling, suggesting that post-translational modifications affecting NF-κB are also redox-sensitive processes (Rahman et al., 2004). In the present study, we observed that the bacterial signaling caused by rVvpM acting through the ROS-PKCα-JNK-NF-κB pathway via lipid-raft-associated ANXA2 is not required for autophagy activation, ensuring that the autophagy-mediated activation of the NLRP3 inflammasome is independent of the action of lipid rafts. Thus, our data also provide important evidence that VvpM induces the transcriptional regulation of NF-κB as well as the hypomethylation of the *IL-1*β promoter to promote IL-1β production coupled with necrotic macrophage death.

On the other hand, our results reveal that VvpM acting through non-lipid raft ANXA2 promotes autophagic death and inflammasome-dependent IL-1β secretion in macrophages. Although the role of non-lipid rafts coupled with ANXA2 has not been identified, the non-lipid raft protein transferrin receptor critically participates in promoting bacterial signaling of *Neisseriaceae* and *Pasteurellaceae* families (Moraes et al., 2009). While autophagic cell death is an essential innate immune signaling mechanism to promote cytokine and chemokine production, and bacterial clearance mechanisms (Xu et al., 2006; Labbe and Saleh, 2008; Kirienko et al., 2015), many pathogens, including *S. typhimurium, M. tuberculosis* and *L. monocytogenes*, are reported to manipulate the autophagosome machinery for their replication and control their phagocytosis by using immune cells to facilitate their dissemination into the blood stream (Birmingham et al., 2006, 2007; Biswas et al., 2008; Cemma and Brumell, 2012). Thus, describing the processes in autophagy-related events may reveal why certain cells may be more or less susceptible to pathogen-induced cell death and may reveal novel therapeutic targets. Importantly, rVvpM acting on non-raft ANXA2 distinctively regulates autophagic cell death in macrophages via Atg5, which is critical for the formation of autophagosomes. Moreover, Atg5 covalently conjugates with Atg12 and interacts with Atg16L1 to form a dimeric complex known as the Atg16L1 complex which promotes LC3 lipidation in expanding phagophores for elongation of the autophagic membrane (Ohsumi, 2001; Geng and Klionsky, 2008). In addition to Atg5, recent research employing the ANXA2 KO mouse model has provided new insights into the biological functions of ANXA2, indicating that ANXA2 is a critical autophagy inducer for the recruitment of phosphatidylserine and phosphatidylinositides into Atg16L-positive vesicles, representing very early events in the generation of phagophores (Morozova et al., 2015). The formation of autophagophore is also related to the ability of ANXA2 to interact and organize cytoskeleton proteins including actins and actin nucleation factor Spire1 and Arp2/3 located in both non-lipid and lipid raft for inward budding (Hayes et al., 2009; Morozova et al., 2015). Thus, it is possible that non-lipid ANXA2 could binds to actin-related proteins to form the autophagophore in rVvpM-treated macrophage. Hence, the results of our present study indicate that rVvpM has unique infectious stratagems by which to initiate Atg5-dependent autophagic cell death via the non-lipid raft ANXA2 and that this bacterial signaling pathway acts in concert with the lipid raft-associated pathways in manipulating IL-1β production. Although previous reports have long considered that the autophagy pathway prevents tissue inflammation through its role in apoptotic cell corpse clearance, our results clearly indicate that VvpM mediates the inflammatory response of *V. vulnificus* by promoting autophagic cell death. A remaining question is how does VvpM utilize the inflammatory signaling pathway via autophagy. Given that inflammasome activation is an alternative cell death pathway associated with releasing mature pro-inflammatory cytokine IL-1β (Lamkanfi et al., 2011; Guo et al., 2015), our present results reveal that VvpM is a major virulence factor of *V. vulnificus* and is responsible for triggering the activation of caspase-1, which is cleaved by the assembly of the NLR protein NLRP3 and the ASC adaptor to promote IL-1β production, and, importantly, that the NLRP3 inflammasome assembly strongly depends on autophagy activation. These results are consistent with those in previous studies showing that autophagy controls inflammasome-dependent IL-1β secretion via Atg5, suggesting a role of autophagy in the positive regulation of inflammasome activation (Dupont et al., 2011).

During the early stages of the VvpM treatment (for 6 h), we observed that VvpM had the ability to promote the NLRP3 inflammasome complex and ASC speck formation without affecting protein expression levels, whereas later in the treatment (for 24 h), VvpM clearly induced expressions of NLRP3, caspase-1, and IL-1β, which are all associated with the formation of the NLRP3 inflammasome complex. These results indicate that genes related to NLRP3 inflammasomes must be transcriptionally induced by some key mediator of immunity to generate IL-1β production continually. Although we did not investigate the reasons for the unexpected contrary results regarding the up-regulation of Tnf expression and down-regulation of Ctsb, Ccl12, and Cxcl3, we did note that VvpM has the additional ability to stimulate the expressions of JNK, IKK-β, and Traf6, which are responsible for NF-κB activation (Matsumura et al., 2003; Lamkanfi et al., 2004; Sollberger et al., 2014; Lee et al., 2015c) and TGF-β activated kinase 1 (TAK1) and CCL7 expression, all of which are related to NLRP3 inflammasome activation (Inoue et al., 2012; Latz et al., 2013). These results suggest that VvpM stimulates IL-1β production coupled with necrotic macrophage death by regulating the Atg5-depednent formation of the NLRP3 inflammasome complex and by governing expressions of genes closely related to NLRP3 inflammasomes. Taken together, these findings therefore indicate that VvpM acting with non-raft ANXA2 is a major signaling regulator of autophagy in promoting NLRP3 inflammasome activation, although earlier studies found a mutual relationship between autophagy and inflammasomes (Yuk and Jo, 2013). Concerning the role of RTX in necrotic cell death, it is well-known multifunctional exotoxins of *V. vulnificus* and causes the necrotic cell death and/or IL-1β production through the lysis of a variety of cell types including macrophages (Toma et al., 2010) and epithelial cells (Lee et al., 2007; Kim et al., 2013). Moreover it already has been reported to play a major role in the pathogenesis of *V. vulnificus* (Toma et al., 2010; Jeong and Satchell, 2012; Kim et al., 2013). Given current *in vitro* observations that RTX is also responsible for ROS production, PKC phosphorylation and IL-1β production, it is possible that RTX induces necrotic cell death. However, it recently have shown that RTX has multiple effector domains, which catalyse the site-specific processing of the Switch I region of Ras and Rap1 (Antic et al., 2015). Thus, we suggest that the virulence mechanism of RTX during the necrotic cell death differs from the VvpM, which efficiently regulates two pathogenic pathways via distinct spatial targeting by membrane ANXA2.

Finally, in mouse models of *V. vulnificus* infection, our results obtained from gain- and loss-of-function approaches for VvpM revealed that the functional importance of two pathogenic pathways via ANXA2 to promote IL-1β production coupled with macrophage death may contribute to the colonization of *V. vulnificus* in the gut. Our results are consistent with those in a previous report showing that *V. vulnificus* not only induces massive inflammation, leading to the recruitment of monocytes, neutrophils and F4/80-positive macrophages (Jeong and Satchell, 2012), but that it also kills phagocytes in the gut (Toma et al., 2010; Lo et al., 2011). Concerning the role of VvpM in intestinal colonization, it is unclear whether the functional role of VvpM in facilitating *V. vulnificus* colonization is a direct effect of its capacity to grow in the microenvironment of the small intestine or, alternatively, a sequential process involving other cellular signaling events related to the killing of the recruited phagocytes. In agreement with our results, it was previously shown that *Salmonella* induces pyroptosis, leading to inflammation, which allows *Salmonella* to use tetrathionate respiration in the promotion of bacterial growth and colonization (Fink and Cookson, 2007). Thus, it is possible that the killing of macrophages with VvpM together with the promotion of the IL-1β production is one mechanism that would promote rapid *in vivo V. vulnificus* growth and colonization. We also used an ileal-ligated mouse model to investigate the actual contribution of VvpM to inflammatory responses under similar infection levels of WT, *VvpM* mutant, and complementation. Our data revealed that VvpM is required for the initiation of massive necrotizing inflammation, accompanying the activation of autophagy, JNK/NF-κB, caspase-1, and IL-1β. While our results indicate that the action of VvpM against infiltrated phagocytes is induction of pro-inflammatory responses, our results do not negate the contention that this toxin has a significant role in the intestinal epithelium, just as it does in Caco-2 cells and in the findings in previous reports (Lee M. A. et al., 2014; Lee et al., 2015a).

In conclusion, our results suggest that VvpM induces two different pathogenic pathways closely related to IL-1β production coupled with necrotic macrophage death and that VvpM regulates the NF-κB-dependent IL-1β production pathway via lipid-raft-dependent ANXA2 recruitment, whereas VvpM acting on non-lipid raft ANXA2 additively facilitates the activation of NLRP3 inflammasome via the Atg5-dependent autophagic pathway (Figure 8F). Given the current *in vivo* observations, we further suggest that VvpM is a new virulence factor responsible for regulation of the intestinal colonization of *V. vulnificus*. Thus, research highlighting the rVvpM signaling pathways involved in IL-1β production coupled with necrotic macrophage death may indicate potential therapeutic targets for strategic modulation of *V. vulnificus* infections.

## Author contributions

Study concept and design, acquisition of data, analysis and interpretation of data, statistical analysis, and drafting of the manuscript: SL and HJH. Acquisition of data, analysis and interpretation of data: YHJ, JSK, HJL, SHL, and KKJ. Technical or material support, analysis and interpretation of data: KL and SHC.

### Conflict of interest statement

The authors declare that the research was conducted in the absence of any commercial or financial relationships that could be construed as a potential conflict of interest.
