# Supplementary material for: A Vibrio vulnificus VvpM Induces IL-1β Production Coupled with Necrotic Macrophage Death via Distinct Spatial Targeting by ANXA2
Source: Front Cell Infect Microbiol. 2017 Aug 11;7:352. doi: 10.3389/fcimb.2017.00352 (PMC5554522; doi:10.3389/fcimb.2017.00352)
Supplement: Supplementary file 1 [file DataSheet1.PDF]

Table S1. Oligonucleotides used in this study

Name	Oligonucleotide Sequence (5' → 3')a, b	Use
**For mutant construction**		
VVHBA01-F	ATGCTTAATAACAAAAATAGAAATGTAGGACG	Deletion of *vvhBA* ORF
VVHBA01-R	AGTGGATCCCTCAGATTGGAAGCG	
VVHBA02-F	AGGGATCCACTGTTTGAAGCGGAAG	Deletion of *vvhBA* ORF
VVHBA02-R	CTAGAGTTTGACTTGTTGTAATGTGGGTTTC	
RTXAN5	TACGGCAACATTTCCGTGCACAAAG	Deletion of *rtxA* ORF
RTXAN3	AACGGGATCCCGCGAATATTCCTAG	
RTXAC5	CGGGATCCCGTTTAACTTTGCTGTG	Deletion of *rtxA* ORF
RTXAC3	ACCGTTGAATTGACTGAACTTAGAG	
vvpM-upF	CGTGACATTTTGGGCCCTCTAGT	Deletion of *vvpM* ORF
vvpM-upR	CGGGATCCCCGACATCATGGGCACCAACAT	
vvpM-downF	CGGGATCCCATTTTGTTCCCACCATAGG	Deletion of *vvpM* ORF
vvpM-downR	CGAGCTCGGTGAACCTTAAGATGGCGC	
**For mutant complementation**		
VVPM001F	GGATCCAGTTTTGCAATCCTATGGTG	Amplification of the *vvpM* ORF
VVPM001R	GAGCTCCTAGTTTTGTGAACAAAAGG	

[a] The oligonucleotides were designed using the *V. vulnificus* MO6-24/O genomic sequence (GenBank™ accession number CP002469 and CP002470, www.ncbi.nlm.nih.gov).

[b] Regions of oligonucleotides not complementary to the corresponding genes are underlined.

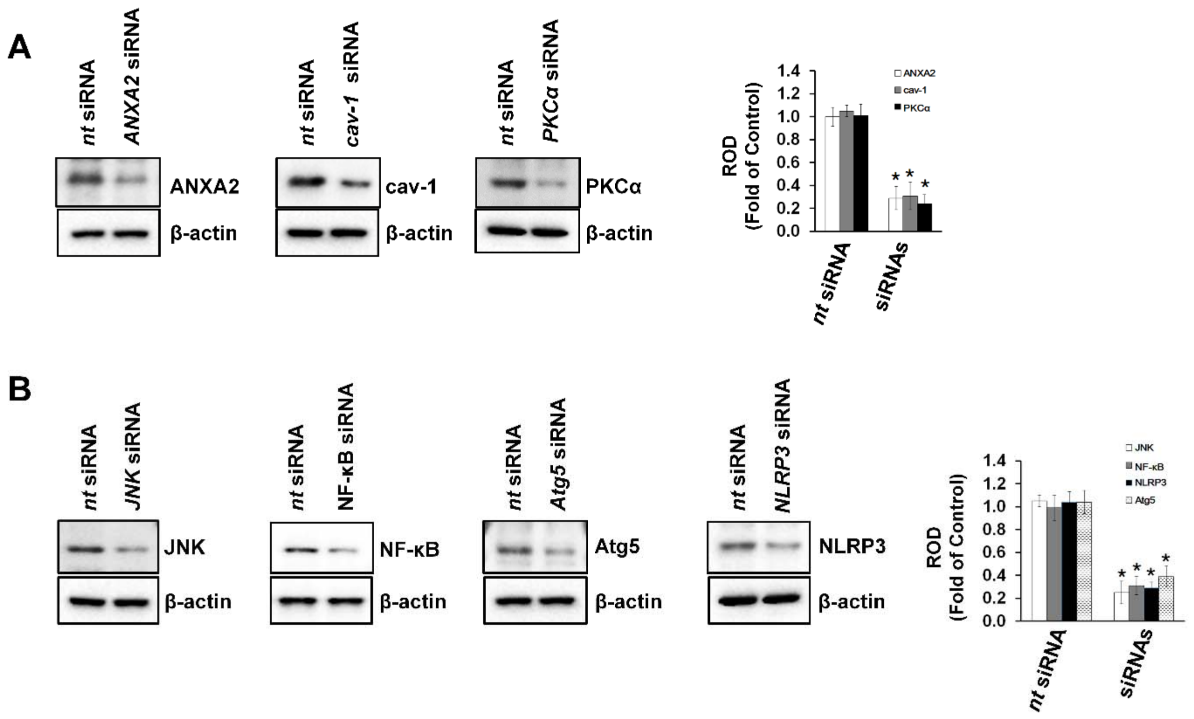

Figure S1. **Effect of siRNA on target proteins.** Cells were transfected for 36 h with *ANXA2, cav-1, PKCα, JNK, NF-κBp65, Atg5,* and *NLRP3* or *non-targeting* (*nt*) siRNA using HiPerFect Transfection Reagent. Protein expressions were analyzed by using Western blot. The siRNA efficacies for NXA2, cav-1, PKCα, JNK, NF-κBp65, Atg5, and NLRP3 determined by Western blot were 71, 69, 76, 75, 69, 71, and 61%, respectively Error bars represent the means ± S.E. from three independent experiments. *, $P < 0.05$ vs *nt* siRNA.

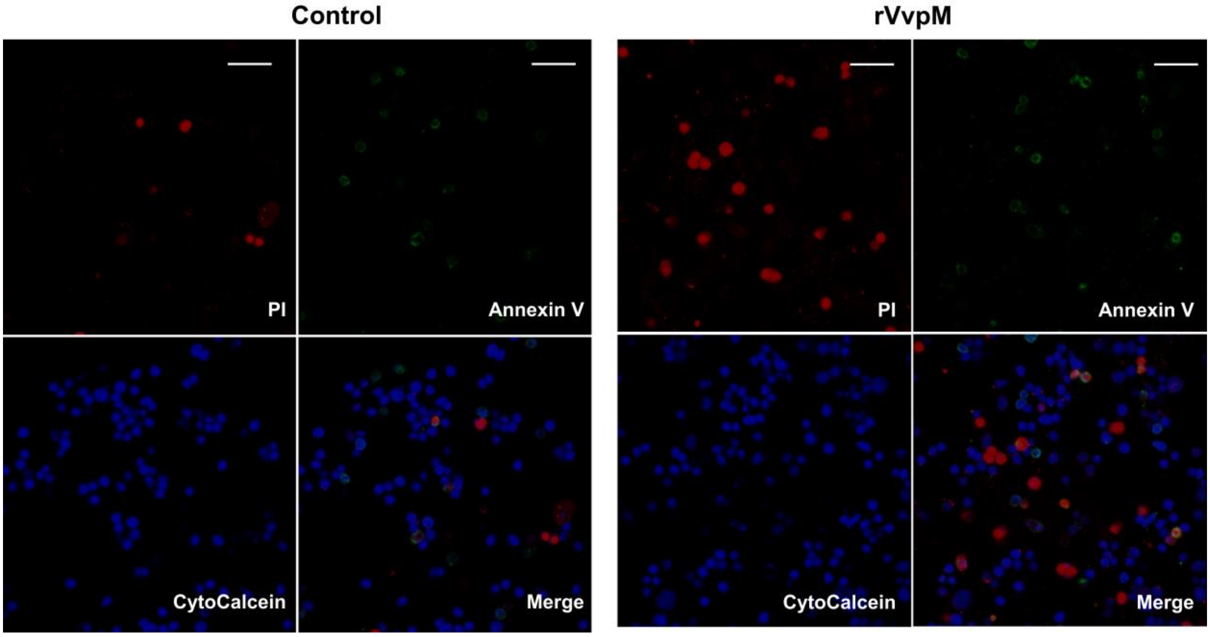

Figure S2. **The role of rVvpM in the promoting of cell death.** Raw 264.7 cells were incubated with 100 pg/mL of rVvpM for 24 h. Cell death induced by rVvpM were determined by staining with annexin V for apoptotic cells (green), PI for necrotic cells (red), and CytoCalcein for live cells (blue). Scale bars, 100 μm. *n* = 3.

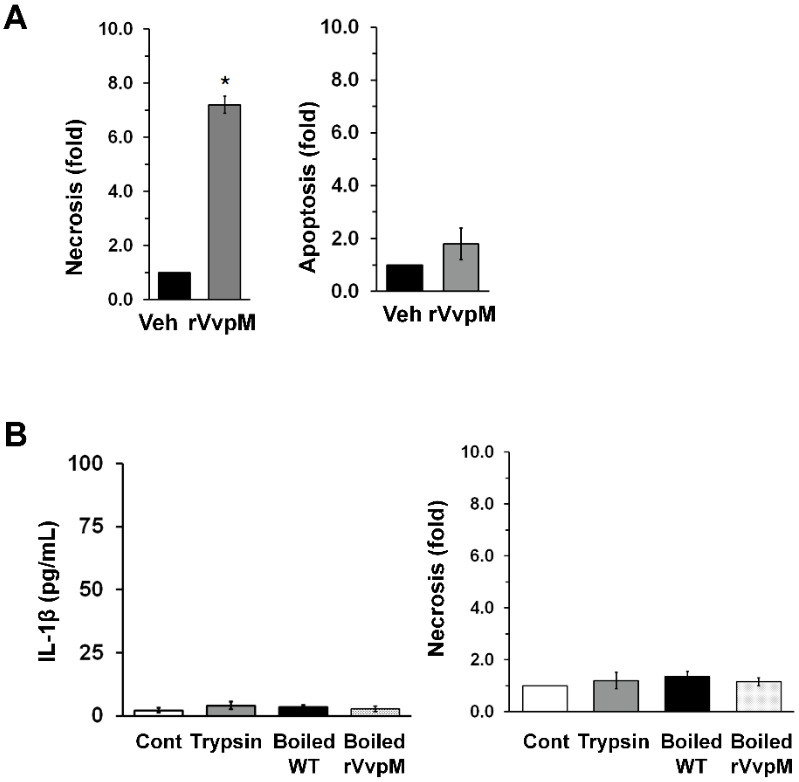

Figure S3. **The role of rVvpM in the promoting of cell death.** Raw 264.7 cells were incubated with 100 pg/mL of rVvpM for 24 h. (A) Quantitative analysis of the fold changes of apoptotic and necrotic cells detected by 7-aminoactinomycin D is shown. Error bars represent the means ± S.E. ($n = 3$). *, $P < 0.05$ versus Veh (boiled rVvpM). (B) The levels of IL-1β protein and necrosis in cell treated with Cont (PBS), 100 pg/mL of trypsin, boiled WT, and boiled rVvpM for 24 h were quantified by ELISA and Apoptosis/ Necrosis Detection kit, respectively. $n = 3$.

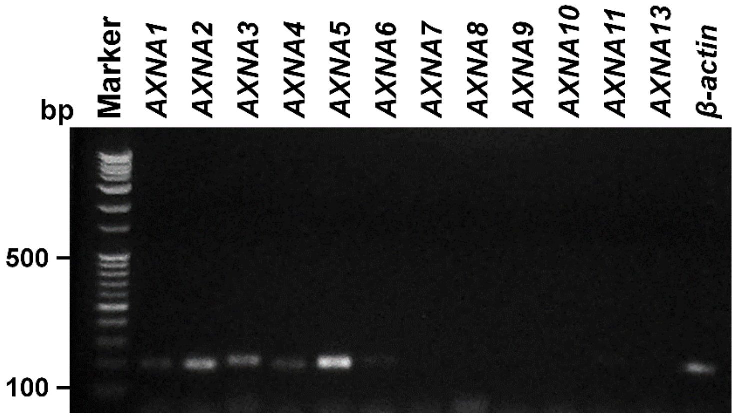

Figure S4. **Expression of *Annexins (ANXA)* mRNAs in Raw 264.7 cells.** A representative 1% agarose gel following RT-PCR is shown. *n* = 3.

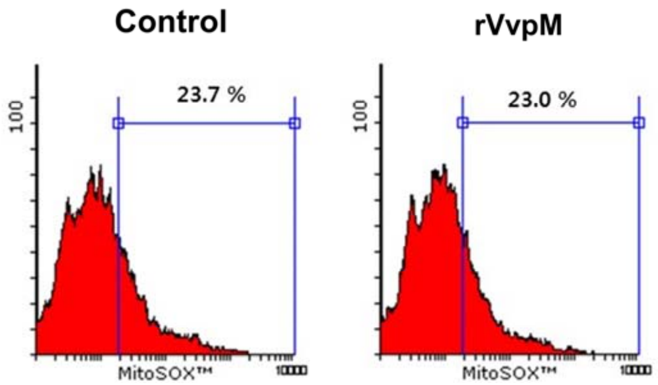

Figure S5. **The role of rVvpM in regulation of mitochondrial ROS production.** Raw 264.7 cells were incubated with 100 pg/mL of rVvpM for 30 min. Mitochondrial ROS production was measured by flow cytometry following staining with MitoSOX ™ Red. The percentages of flow cytometric data indicate the cells strongly stained with MitoSOX ™ Red at consistent threshold. *n* = 4.

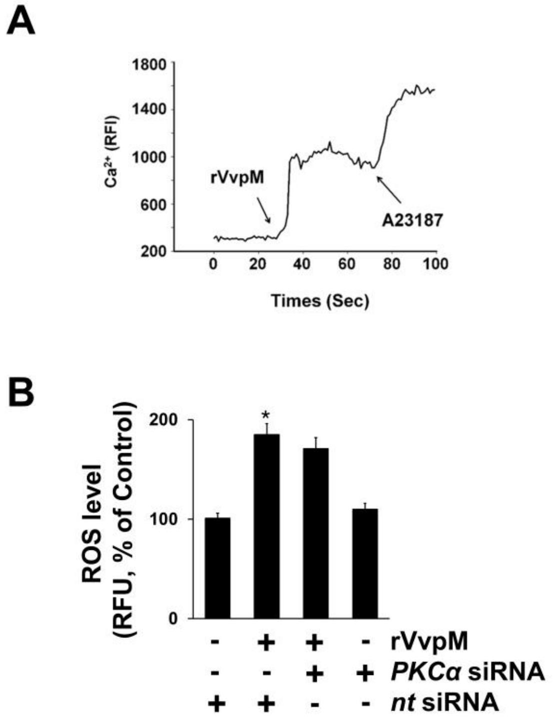

Figure S6. rVvpM regulates calcium influx and ROS production. (A) A calcium influx in cells treated with rVvpM**.** Changes in [Ca^{2+}]i were monitored by confocal microscopy, and data are expressed as relative fluorescence intensity (RFI, F/F0 %, arbitrary unit). A23187 (10 μM) was used as a positive control. $n = 4$. (B) Cells were transfected with siRNAs for *PKCα* for 24 h prior to rVvpM exposure for 30 min. ROS production is shown. Data represent the mean ± S.E. n = 3. *, $P < 0.01$ versus *nt* siRNA. RFU, relative fluorescence units.

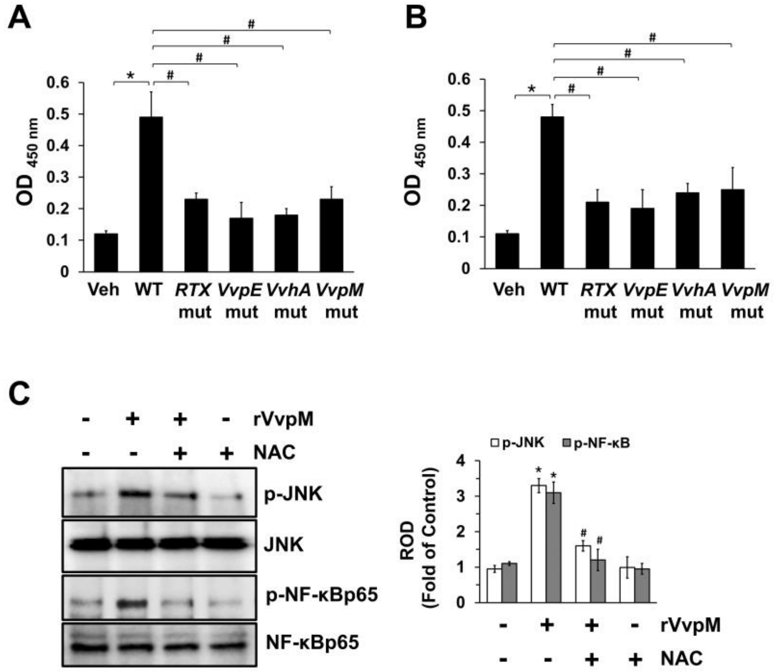

Figure S7. The role of *V. vulnificus* in regulation of JNK and NF-κBp65.

The DNA binding activity of NF-κBp65 in Raw 264.7 (A) and Caco-2 cells (B) infected with WT or various mutant deficients was determined by using NF-κB p65 transcription assay kit. The competitor dsDNA was used negative control. $n = 3$. *, $P < 0.01$ versus Veh (boiled WT). $^{\#}$, $P < 0.01$ versus WT. ROD, relative optical density. (C) Cells were pre-treated with NAC (10 μM) for 30 min prior to rVvpM exposure. Data represent the mean ± S.E. n = 3. *, $P < 0.01$ versus cells with no treatment. $^{\#}$, $P < 0.05$ versus rVvpM. ROD, relative optical density.

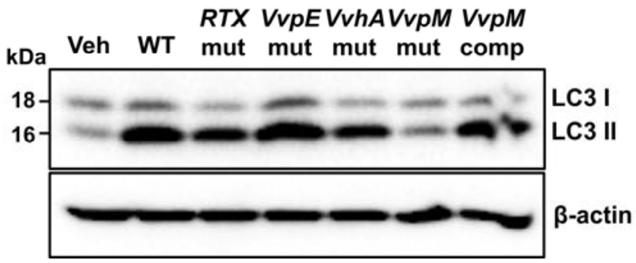

Figure S8. **The role of *V. vulnificus* in regulation of LC3 expression.** The expressions of LC3 I and II in a cells infected with WT, various mutant deficient, or complemented VvpM mutant for 1 h are shown. *n* = 4.

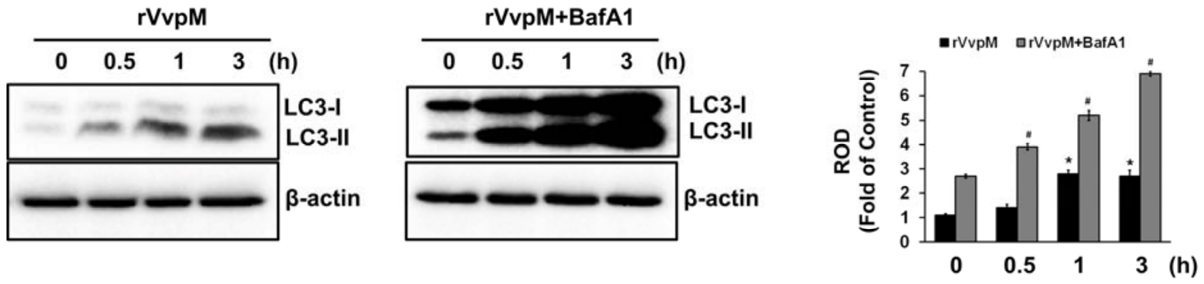

Figure S9. **rVvpM induced autophagy, which is independent of lysosomal membrane permeabilization.** Cells were incubated in the presence or absence of 10 nM BafA1 for 60 min prior to rVvpM exposure. Time-dependent changes in the level of LC3 expression is shown. Data represent mean ± S.E. n = 3. *, P < 0.01 versus cells with no treatment, $^{\#}$, P < 0.01 versus BafA1 alone. ROD, relative optical density

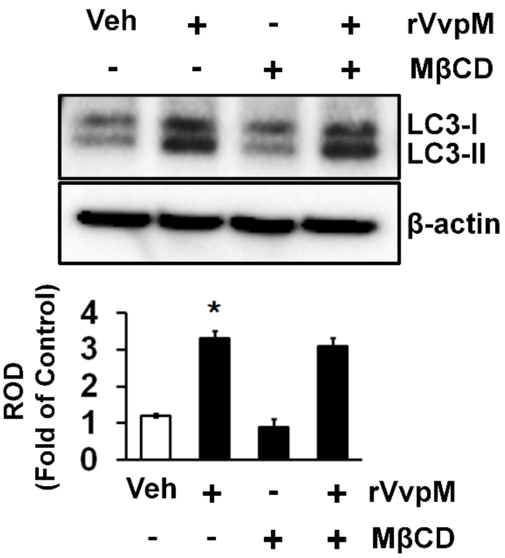

Figure S10. **LC3 expression induced by rVvpM is an independent of the action of lipid rafts.** Cells were pre-treated with MβCD (0.1 mM) for 60 min prior to rVvpM exposure for 3 h. The expression level of LC3 expression was determined by Western blot. $n = 3$. *, $P < 0.05$ versus Veh (boiled rVvpM). ROD, relative optical density.

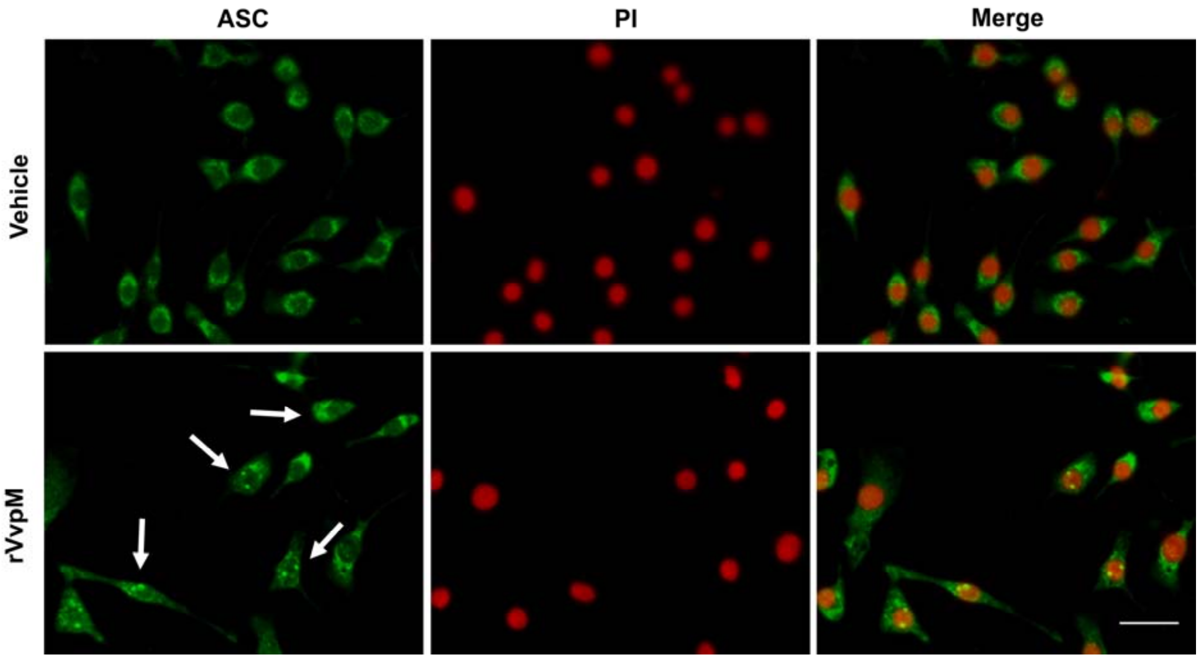

Figure S11. **rVvpM induces ASC speck formation.** Raw 264.7 cells were incubated with 100 pg/mL of rVvpM for 6 h. The speck formation of ASC by rVvpM were determined by staining with ASC (green) and PI (red). Scale bars, 100 µm. *n* = 3.
